# Effect of Polymorphism on the Sorption Properties of a Flexible Square-Lattice Topology Coordination Network

**DOI:** 10.1021/acsami.4c03777

**Published:** 2024-04-26

**Authors:** Aizhamal Subanbekova, Andrey A. Bezrukov, Volodymyr Bon, Varvara I. Nikolayenko, Kyriaki Koupepidou, Debobroto Sensharma, Sousa Javan Nikkhah, Shi-Qiang Wang, Stefan Kaskel, Matthias Vandichel, Michael J. Zaworotko

**Affiliations:** † Department of Chemical Sciences, Bernal Institute, University of Limerick, Limerick V94 T9PX, Republic of Ireland; ‡ Faculty of Chemistry, Technische Universität Dresden, Bergstrasse 66, Dresden 01062, Germany; § Institute of Materials Research and Engineering (IMRE), Agency for Science, Technology and Research (A*STAR), 2 Fusionopolis Way, Singapore 138634, Singapore

**Keywords:** crystal engineering, polymorphs, metal−organic frameworks, structural transformation, gate opening, *in situ* PXRD

## Abstract

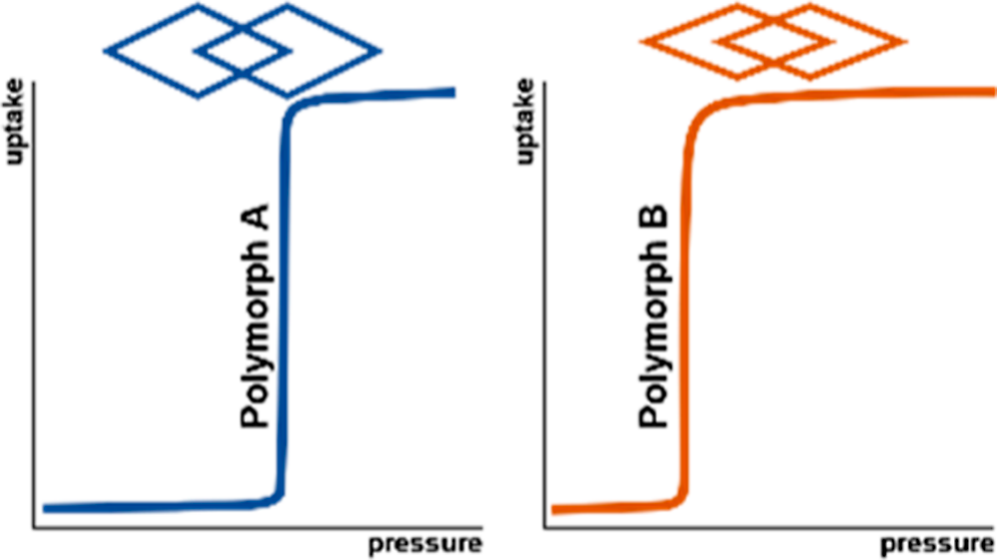

The stimulus-responsive behavior of coordination networks (CNs), which switch between closed (nonporous) and open (porous) phases, is of interest because of its potential utility in gas storage and separation. Herein, we report two polymorphs of a new square-lattice (**sql**) topology CN, **X-sql-1-Cu**, of formula [Cu(Imibz)_2_]_*n*_ (HImibz = {[4-(1*H*-imidazol-1-yl)phenylimino]methyl}benzoic acid), isolated from the as-synthesized CN **X-sql-1-Cu-(MeOH)**_**2**_**·2MeOH**, which subsequently transformed to a narrow pore solvate, **X-sql-1-Cu-A·MeOH**, upon mild activation (drying in air or heating at 333 K under nitrogen). **X-sql-1-Cu-A·MeOH** contains MeOH in cavities, which was removed through exposure to vacuum for 2 h, yielding the nonporous (closed) phase **X-sql-1-Cu-A**. In contrast, a more dense polymorph, **X-sql-1-Cu-B**, was obtained by exposing **X-sql-1-Cu-(MeOH)**_**2**_**·2MeOH** directly to vacuum for 2 h. Gas sorption studies conducted on **X-sql-1-Cu-A** and **X-sql-1-Cu-B** revealed different switching behaviors to two open phases (**X-sql-1-Cu·CO**_**2**_ and **X-sql-1-Cu·C**_**2**_**H**_**2**_), with different gate-opening threshold pressures for CO_2_ at 195 K and C_2_H_2_ at 278 K. Coincident CO_2_ sorption and *in situ* powder X-ray diffraction studies at 195 K revealed that **X-sql-1-Cu-A** transformed to **X-sql-1-Cu-B** after the first sorption cycle and that the CO_2_-induced switching transformation was thereafter reversible. The results presented herein provide insights into the relationship between two polymorphs of a CN and the effect of polymorphism upon gas sorption properties. To the best of our knowledge, whereas **sql** networks such as **X-sql-1-Cu** are widely studied in terms of their structural and sorption properties, this study represents only the second example of an in-depth study of the sorption properties of polymorphic **sql** networks.

## Introduction

Metal–organic materials (MOMs),^1^ including porous coordination polymers^2^ such as metal–organic frameworks (MOFs),^3^ have attracted attention due to the tunability of their composition and properties through crystal engineering approaches,^1^ which have enabled the development of MOMs with potential utility for gas and vapor storage/separation,^4,5^ drug delivery,^6^ catalysis,^7^ and chemical sensing.^8^ Of more than 118,000 structures deposited in the MOF subset of the Cambridge Structural Database (CSD),^9^ the majority are characterized as rigid MOFs.^9−11^ Rigid MOFs, also known as second-generation physisorbents,^12^ are stable upon guest solvent removal and typically show fixed pore volume and type I gas sorption isotherm profiles.^12^ A limitation of type I isotherms is that the gas uptake at relevant desorption pressures results in decreased working capacity for gas storage applications.^13^

An alternative to rigid materials is exemplified by a third class of physisorbents, known as flexible metal–organic materials (FMOMs) or soft porous crystals.^12,14^ FMOMs include porous coordination polymers that undergo structural changes triggered by external stimuli such as pressure.^14,15^ Such structural transformations can involve “switching” between closed (nonporous) and open (porous) phases by “gate-opening” events over a narrow pressure range as characterized by stepped or type F-IV isotherms.^16^ This isotherm profile means that switching FMOMs offer potential utility in gas storage, as they can enable higher working capacities compared to rigid MOMs.^17,18^ Controlling the gate-opening pressure of FMOM platforms is critical for both storage and separation applications; various crystal engineering strategies have been studied in this context, including linker substitution,^19−22^ metal substitution,^23−25^ and packing polymorphism.^26^ Polymorphism, the existence of two or more different crystal packing motifs for the same compound,^27^ has been studied in pharmaceutical science and molecular solids for decades^27^ but remains understudied in MOFs.^28^ Nevertheless, it is recognized that polymorphs can impact physical properties such as pore volume,^29,30^ thermal stability,^31−33^ and adsorption profiles.^26,34^

Herein, we address the impact of polymorphism in a 2D coordination network (CN) with square lattice (**sql**) topology. **sql** is one of the most common topologies found in MOFs, with more than 9000 **sql** networks archived in the TOPOS TTO ∩ CSD database.^35,36^ MOMs with **sql** topology offer high modularity^37^ and potential for clay-like layer expansion/contraction in response to external stimuli.^38^ MOMs with **sql** topology such as **UTSA-300**, **NCU-100**, **ZUL-210**, and **sql-16-Cu-NO**_**3**_(39,40) have exhibited benchmark properties in the context of gas separations. In 2001, Li and Kaneko introduced the prototypal switching **sql** network, **ELM-11**, or [Cu(bpy)_2_(BF_4_)_2_],^41^ for which type F-IV^16^ adsorption isotherms were recorded as a result of layer expansion induced by exposure to N_2_, CO_2_, or Ar. An **ELM-11** variant, **sql-1-Co-NCS**, was reported to exhibit benchmark separation of *o*-xylene from a C8 aromatic mixture.^42^**sql** networks can also be designed using a mixed-linker approach, as exemplified by **sql-1,3-Co-NCS**, comprising two N-donor linkers.^43^ Recent publications from our group have explored mixed-linker **sql** networks based on N-donor and O-donor linkers, resulting in water-induced structural transformations.^35,44^**sql** networks can also be designed using a single linker containing both N- and O-donor functionality (bifunctional linker). Such linkers typically afford CNs with **dia** and **sql** topologies of formula ML_2_ (M = divalent metal cation and L = ditopic linker ligand).^45^ To the best of our knowledge, the first ML_2_ network based on a bifunctional linker was l-phenylalanine reported by Enwall *et al.* in 1971,^46^ while recent examples include [**Cu(Qc)**_**2**_] as reported by Chen *et al.*(47) According to a recent survey, this type of **sql** net based upon bifunctional linkers is relatively understudied compared to other types.^43^

**sql** networks remain underexplored with respect to polymorphism. Our analysis of the TOPOS TTO ∩ CSD database^36,48^ revealed only 37 examples of polymorphism in **sql** networks (Figure S1, Table S1). Furthermore, polymorphism was mentioned by the authors in less than half of these cases,^49−66^ with most examples discovered through separate studies that did not address comparison of properties. In a recent study, we demonstrated that packing polymorphism (AA *vs* AB) can profoundly impact hydrocarbon separation performance in **sql-NbOFFIVE-bpe-Cu**.^26^ To our knowledge, this report represented the second in-depth investigation of the impact of polymorphism on the adsorption properties of **sql** networks. Herein, we report on polymorphism and its impact upon sorption properties in a new ML_2_ network with **sql** topology, **X-sql-1-Cu**, Cu(Imibz)_2_, HImibz = {[4-(1*H*-imidazol-1-yl)phenylimino]methyl}benzoic acid.^67^ HImibz was first reported as a linker in **X-dia-2-Cd**.^68^ The synthesis, characterization, and relationship between two polymorphs of **X-sql-1-Cu**, **X-sql-1-Cu-A** and **X-sql-1-Cu-B**, are addressed herein using single-crystal X-ray diffraction (SCXRD), gas sorption, and coincident *in situ* powder X-ray diffraction (PXRD).

## Experimental Section

All reagents and solvents were procured from Sigma-Aldrich and used without further purification. HImibz was synthesized using a modified procedure [see Supporting Information for detailed synthetic procedure and characterization of the linker (Figures S2 and S3)].^67^ A new batch of **X-sql-1-Cu-A** was prepared for each sorption analysis due to its tendency to undergo phase transformation to **X-sql-1-Cu-B**. SCXRD data were collected on a Bruker Quest diffractometer equipped with a CMOS detector and IμS microfocus X-ray source (Cu Kα, λ = 1.5406 Å) under N_2_ flow at 100 K. PXRD experiments were conducted using microcrystalline samples on a PANalytical Empyrean diffractometer (40 kV, 40 mA, Cu Kα1,2, λ = 1.5406 Å) in Bragg–Brentano geometry. Variable-temperature PXRD (VT-PXRD) data were recorded using a PANalytical X’Pert Pro-MPD diffractometer equipped with a PIXcel3D detector operating in scanning line detector mode operated at 40 kV and 40 mA with Cu Kα radiation (λα = 1.5406 Å). Thermogravimetric analysis (TGA) studies were conducted using a TA Instruments Q50 at a rate of 10 K min^–1^ from 298 to 773 K under a continuous flow of N_2_. Volumetric gas sorption experiments were performed using a Micrometrics 3Flex surface area and pore size analyzer 3500. *In situ* PXRD experiments in parallel to gas adsorption were performed on the Empyrean powder X-ray diffractometer (ω–2θ goniometer, K-Alpha1 system) using a customized setup based on an ARS DE-102 closed-cycle helium cryostat.

## Results and Discussion

### X-ray Crystallography and Characterization

**Figure 1:**
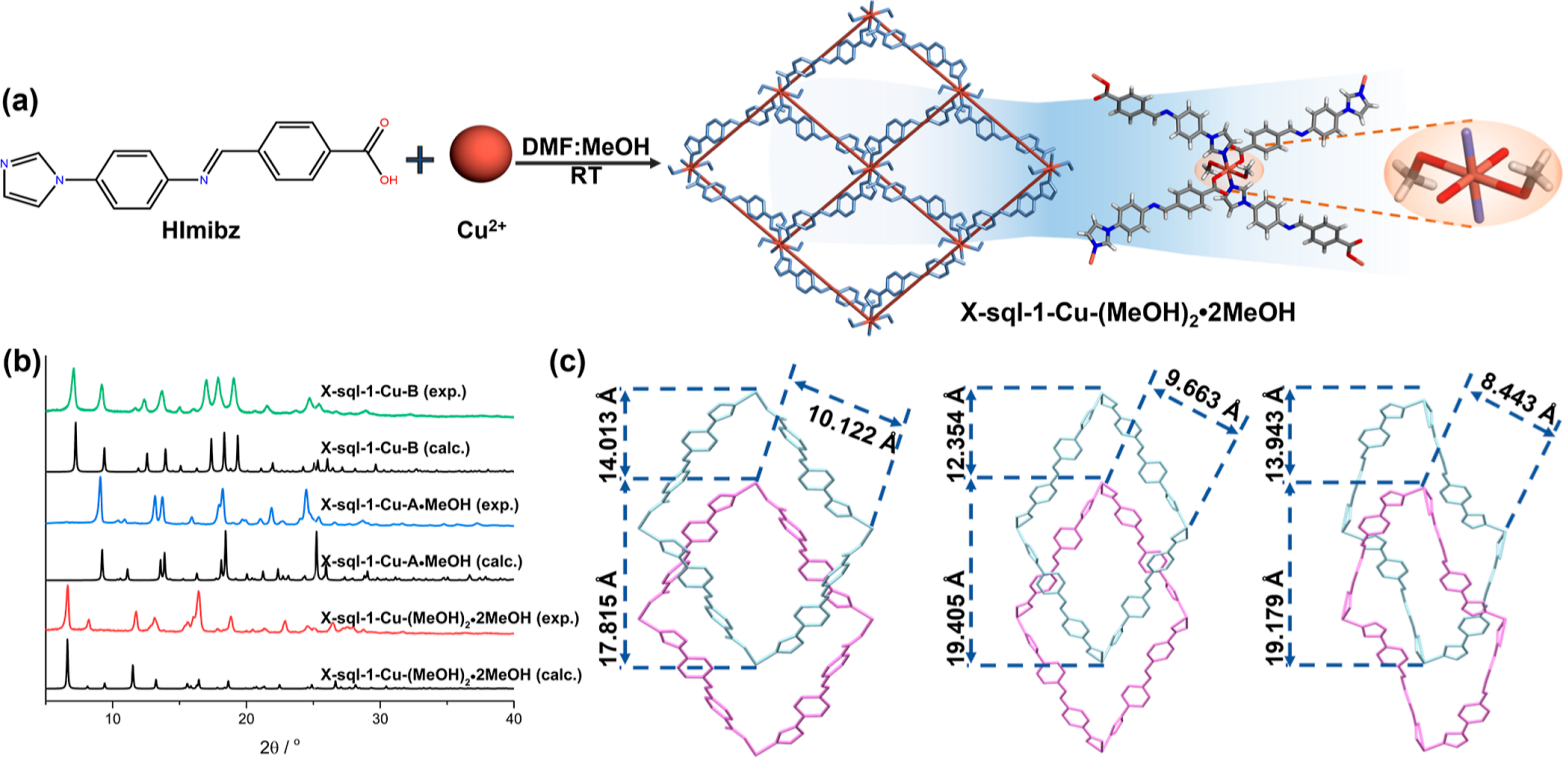
(a) Synthesis of **X-sql-1-Cu-(MeOH)**_**2**_**·2MeOH**; (b) comparison of experimental PXRD patterns with PXRD patterns calculated from SCXRD data; (c) comparison of interlayer distances (from right to left: **X-sql-1-Cu-(MeOH)**_**2**_**·2MeOH**, **X-sql-1-Cu-A·MeOH**, and **X-sql-1-Cu-B**). MeOH = methanol; DMF = *N*,*N*-dimethylformamide.

Single crystals of [Cu(Imibz)_2_(MeOH)_2_]·MeOH, **X-sql-1-Cu-(MeOH)**_**2**_**·2MeOH**, were obtained by layering HImibz and Cu(NO_3_)_2_·3H_2_O in a DMF/MeOH solution at room temperature (Figure 1a). SCXRD revealed that **X-sql-1-Cu-(MeOH)**_**2**_**·2MeOH** had crystallized in the monoclinic space group *P*2_1_/*c* with one Cu^2+^ cation, one Imibz linker, and one coordinated methanol (MeOH) molecule in the asymmetric unit (Table S2). Each Cu^2+^ cation was found to be coordinated to two carboxylate oxygen atoms from two Imibz linkers and two nitrogen atoms from two additional Imibz linkers in the equatorial positions, as well as two MeOH molecules in axial positions (Figure S4, Table S3). This coordination sphere serves as a 4-connected molecular building block to generate a non-interpenetrated 2D **sql** network. A Connolly map^69,70^ of guest accessible volume (1.2 Å probe radius) revealed channels when viewed down [001] that account for 16.9% (318.97 Å^3^) of unit cell volume (Table S2, Figure S5). Whereas not all MeOH solvate molecules in the pores could be crystallographically modeled, the residual electron density, as implemented by SQUEEZE, indicated the presence of 2 methanol solvate molecules per formula unit. TGA showed a weight loss of 18 wt % consistent with two clathrated and two coordinated MeOH molecules per formula unit (calculated 19%) of **X-sql-1-Cu-(MeOH)**_**2**_**·2MeOH** (Figure S6). Solvent loss occurred by 360 K with decomposition starting at 590 K. To expedite synthesis, solvothermal reaction in DMF/MeOH at 333 K was conducted. Calculated and experimental PXRD patterns of **X-sql-1-Cu-(MeOH)**_**2**_**·2MeOH** are in good agreement, indicating bulk phase purity (Figure 1b). For subsequent sorption experiments, samples prepared by the solvothermal method were used.

**Scheme 1:**
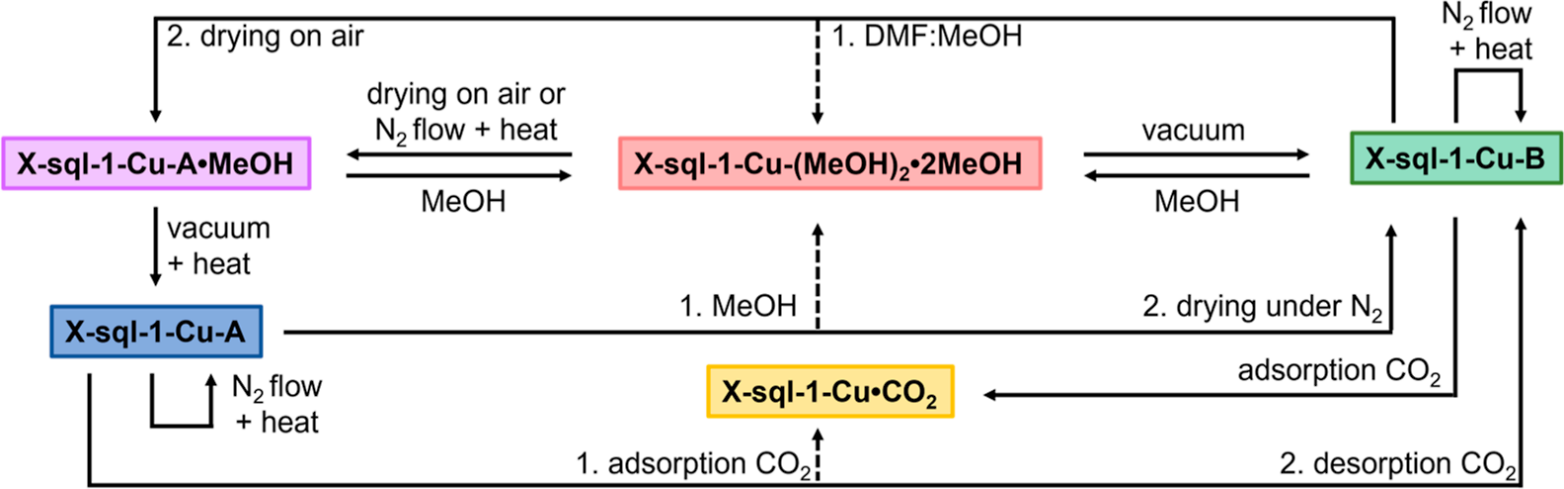
Illustration of the Reversible Phase Transformations of **X-sql-1-Cu-(MeOH)**_**2**_**·2MeOH**, **X-sql-1-Cu-A·MeOH**, **X-sql-1-Cu-A**, and **X-sql-1-Cu-B** The dashed arrow indicates the conversion of one polymorph to another through the solvated **X-sql-1-Cu-(MeOH)**_**2**_**·2MeOH** phase. The “U-shaped” arrow signifies that no changes in structures were observed under these conditions.

**X-sql-1-Cu-(MeOH)**_**2**_**·2MeOH** exposed to ambient air transformed to **X-sql-1-Cu-A·MeOH** (Scheme 1) with PXRD revealing that prominent peaks had shifted to higher 2θ angles (Figure 1b), indicating that **X-sql-1-Cu-A·MeOH** has a smaller unit cell volume. SCXRD data collected at 100 K revealed that, although the space group was unchanged, the coordinated MeOH was replaced by Imibz changing from monodentate to chelate binding (Figure S4, Table S3). This transformation was accompanied by a contraction in the *a* and *b* axes (from 13.72 and 18.80 to 9.68 and 17.06 Å, respectively) and expansion of the *c* axis (from 7.51 to 9.08 Å). The unit cell volume reduced by 21.01%, from 1883.37 to 1486.13 Å^3^. Despite a reduction of guest-accessible volume from 16.9 to 6.7%, one MeOH molecule with partial occupancy was crystallographically located in the cavities of **X-sql-1-Cu-A·MeOH**. This MeOH molecule displayed short contact distances with the imidazole moiety of the host framework (Figure S7). TGA revealed that **X-sql-1-Cu-A·MeOH** lost MeOH below 415 K, with a weight loss corresponding to one MeOH per formula unit (observed 5%, calculated 4.8%). Decomposition occurred at *ca.* 580 K (Figure S6). The experimental and calculated PXRD patterns are consistent (Figure 1c).

Upon heating **X-sql-1-Cu-(MeOH)**_**2**_**·2MeOH** at 333 K under dynamic vacuum, a new phase was observed, **X-sql-1-Cu-B**. PXRD of a bulk sample of **X-sql-1-Cu-B** revealed new peaks at 7.24, 12.58, 123.96, and 17.38° 2θ which are absent in the PXRD pattern of **X-sql-1-Cu-(MeOH)**_**2**_**·2MeOH** (Figure 1b). SCXRD data collected at 100 K revealed that the space group was unchanged (monoclinic *P*2_1_/*c*) but that unit cell parameters had changed to *a* = 12.24 Å, *b* = 14.86 Å, *c* = 8.02 Å, and *V* = 1451.72 Å^3^ (Table S2). VT-PXRD data collected upon **X-sql-1-Cu-(MeOH)**_**2**_**·2MeOH** under dynamic vacuum indicated that the structural transformation to **X-sql-1-Cu-B** was complete at 298 K and remained unchanged up to 473 K (Scheme 1). The calculated and experimental PXRD data are consistent with bulk phase purity (Figure 1b).

Comparison of the single-crystal structures of **X-sql-1-Cu-A·MeOH** and **X-sql-1-Cu-B** revealed that the dihedral angle formed by the benzoate and central phenyl ring of Imibz was 67.21° in **X-sql-1-Cu-A·MeOH***vs* 8.73 and 13.27° in the two disordered components of **X-sql-1-Cu-B** (Figure S8). Rotation and bending of the imidazole and phenyl rings with respect to the central ring of Imibz led to shortening of the diagonal Cu···Cu distance within the **sql** net from 17.06 Å in **X-sql-1-Cu-A·MeOH** to 14.86 Å in **X-sql-1-Cu-B** (Figure S9). **X-sql-1-Cu-B** exhibited a shorter interlayer distance **X-sql-1-Cu-A·MeOH** (8.44 *vs* 9.66 Å), resulting in **X-sql-1-Cu-B** being nonporous (Figure 1c). The SCXRD structures of these three phases are further discussed in more detail in Supporting Information (Figures S8–S10 and Table S4).

Our detailed investigation of phase interconversion in **X-sql-1-Cu** has facilitated the finalization of Scheme 1, summarizing the transitions between all phases through relatively simple conditioning methods (*i.e.*, exposure to CO_2_, MeOH, heat, vacuum, and air). **X-sql-1-Cu-A** and **X-sql-1-Cu-B** were reproducibly isolated from **X-sql-1-Cu-(MeOH)**_**2**_**·2MeOH**. **X-sql-1-Cu-A** was obtained by applying vacuum at 333 K to **X-sql-1-Cu-A·MeOH** for 2 h, a sample of which had been previously prepared by air drying of **X-sql-1-Cu-(MeOH)**_**2**_**·2MeOH** (Figure S11). The scheme illustrates that **X-sql-1-Cu-B** can be isolated along two different pathways: one by applying vacuum to as-synthesized **X-sql-1-Cu-(MeOH)**_**2**_**·2MeOH** (Figure S12) and the other from polymorph **X-sql-1-Cu-A** by resolvating in MeOH and drying under N_2_ flow. Direct conversion of **X-sql-1-Cu-B** to **X-sql-1-Cu-A** is unlikely, with **X-sql-1-Cu-B** being more dense, and we observed that resolvation of **X-sql-1-Cu-B** in MeOH and drying under N_2_ resulted in reversion to **X-sql-1-Cu-B** (Figure S11); conversion of **X-sql-1-Cu-B** back to **X-sql-1-Cu-A** was achieved indirectly by resolvating in DMF/MeOH and drying in air (Figure S13). Thus, the obtained **X-sql-1-Cu-A** was subsequently transformed to **X-sql-1-Cu-B** by exposure to MeOH and drying under N_2_ (Figure S13). Heating of **X-sql-1-Cu-A** or **X-sql-1-Cu-B** under N_2_ to 473 K revealed no phase changes (Figure S14). **X-sql-1-Cu-A** was retained upon cooling to 195 K (Figure S15).

### Relationship between the Various Phases of **X-sql-1-Cu**

#### Polymorphic Conversion upon Desolvation/Solvation

To investigate the effect of MeOH, the MeOH vapor sorption isotherm of activated **X-sql-1-Cu-A** at 298 K was measured. A type F-IV isotherm^16^ with an inflection point of *P*/*P*_0_ = 0.05 indicated transformation of **X-sql-1-Cu-A** to **X-sql-1-Cu-(MeOH)**_**2**_**·2MeOH** with *ca.* 20 wt % saturation uptake (Figure S16). The second and third cycles of MeOH sorption displayed the same uptake but a shift of the inflection point to *P*/*P*_0_ = 0.15. PXRD collected after three cycles of MeOH sorption revealed transformation to the more dense polymorph, **X-sql-1-Cu-B**.

To study the influence of crystal size on polymorph interconvertibility, **X-sql-1-Cu-A** was ground. Scanning electron microscopy (SEM) images revealed that the ground sample had smaller crystal size (10.8 × 6.3 μm^2^) compared to the nonground sample (27.9 × 15.8 μm^2^) (Figures S17–S20, Table S5). The ground sample of **X-sql-1-Cu-A** transformed to **X-sql-1-Cu-B** after MeOH resolvation, resembling the behavior of the nonground sample and suggesting no particle size effect on polymorphic interconvertibility within this size range (Figure S21). Exposure of **X-sql-1-Cu-A** to other coordinating solvents such as ethanol (EtOH) and acetonitrile (MeCN) afforded the same structure as MeOH solvate and formed **X-sql-1-Cu-B** after drying under N_2_ (Figure S22). Conversely, solvation of **X-sql-1-Cu-A** in isopropanol (*i*PrOH) and tetrahydrofuran (THF) triggered transformation to new unidentified phases matching neither **X-sql-1-Cu-A** nor **X-sql-1-Cu-B**, suggesting the existence of more phases of **X-sql-1-Cu** (Figure S22). We attribute this observation to the relatively bulky nature of *i*PrOH and THF which might inhibit the formation of **X-sql-1-Cu-B**. In addition, immersion of **X-sql-1-Cu-A** in dichloromethane did not trigger any transformation.

For further examination of the impact of vapor uptake on polymorphic transformations, a water vapor sorption isotherm was measured at 300 K. This experiment revealed a type I isotherm for **X-sql-1-Cu-A** with *ca.* 5 wt % uptake at 95% relative humidity, while **X-sql-1-Cu-B** displayed negligible uptake of water vapor. Three consecutive cycles of water vapor sorption did not result in structural transformation of **X-sql-1-Cu-A** to **X-sql-1-Cu-B** (Figure S23).

### Gas Sorption

#### Low-Pressure CO_2_ Sorption and *In Situ* Coincident PXRD

The observed structural transformations of **X-sql-1-Cu** triggered by various solvents prompted us to investigate the gas sorption properties of **X-sql-1-Cu**. Activated **X-sql-1-Cu-A** exhibited a type F-IV^16^ CO_2_ isotherm at 195 K which is indicative of switching behavior (Figure S24). The sharp stepped isotherm with an inflection point at *ca.* 0.1 bar resulted in a saturation uptake of 130 cm^3^ g^–1^ (or 5.8 mmol g^–1^), corresponding to *ca.* 3.8 CO_2_ molecules per formula unit. PXRD patterns measured before and after three consecutive CO_2_ adsorption–desorption cycles revealed full conversion of **X-sql-1-Cu-A** to **X-sql-1-Cu-B** (Figure S25).

**Figure 2:**
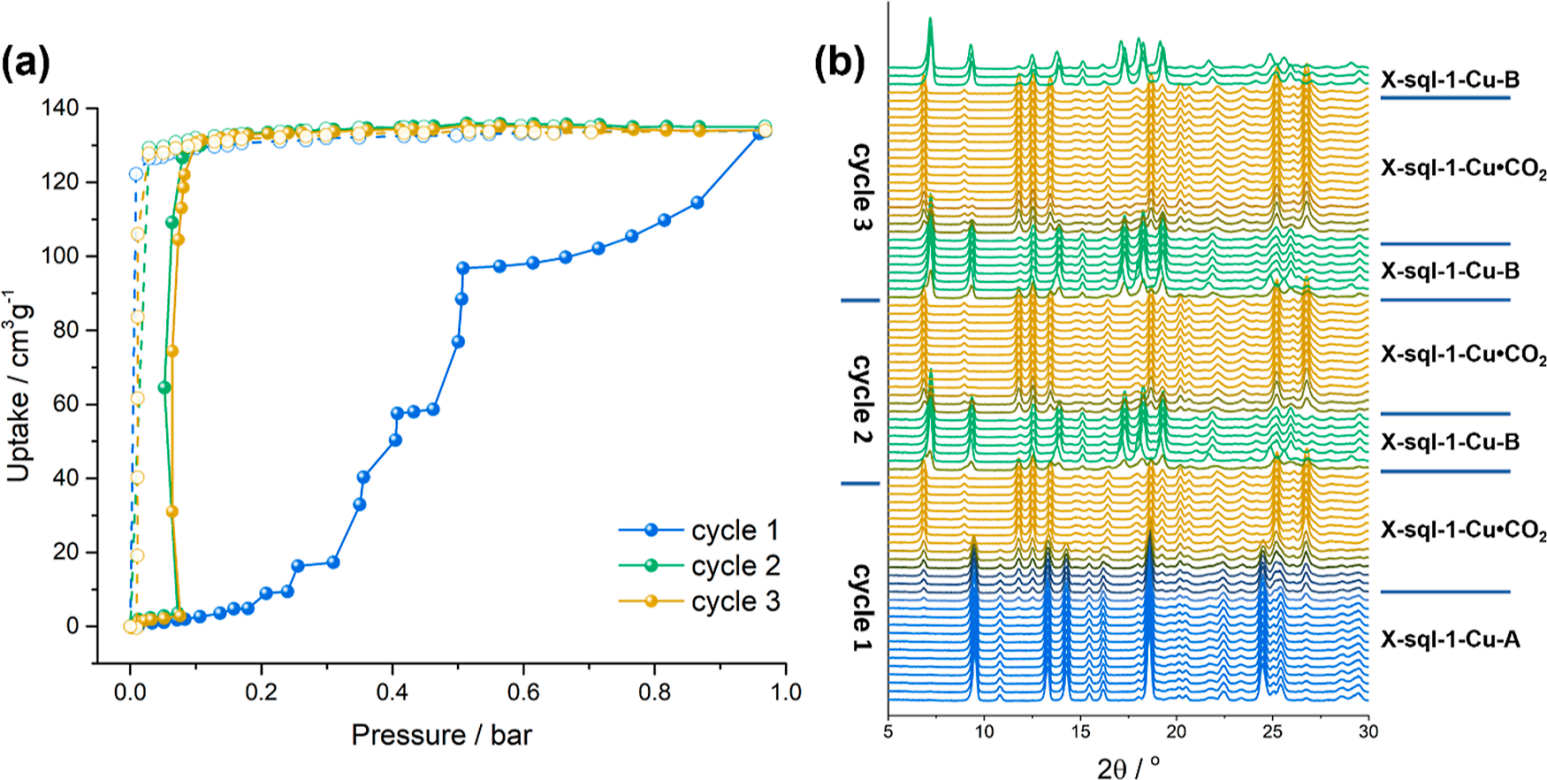
(a) Three cycles of CO_2_ gas sorption collected at 195 K starting from **X-sql-1-Cu-A** polymorph and (b) coincident *in situ* PXRD overlay measured during the adsorption–desorption cycles.

In order to confirm this transformation, three consecutive CO_2_ adsorption–desorption cycles at 195 K were monitored by *in situ* PXRD experiments collected in parallel to sorption, starting from **X-sql-1-Cu-A** (Figure 2). Upon exposure to 0.21 bar of CO_2_, **X-sql-1-Cu-A** transformed to a CO_2_-loaded phase, **X-sql-1-Cu·CO**_**2**_, which was retained until saturation pressure was reached. The PXRD pattern of **X-sql-1-Cu·CO**_**2**_ when compared with **X-sql-1-Cu-A** showed that most peaks had shifted toward lower 2θ angles, indicating a unit cell expansion consistent with the disappearing of peaks at 9.65, 14.2, and 18.5° and appearing of peaks at 6.65, 11.79, and 12.66° (Figure S26). Indexing and Pawley profile fitting of **X-sql-1-Cu·CO**_**2**_ afforded space group *P*2_1_/*c* with unit cell parameters *a* = 13.227(6) Å, *b* = 15.333(15) Å, *c* = 8.817(3) Å, β = 101.74(15)°, and *V* = 1750.6(3) Å^3^ (Figure S27). *In situ* PXRD patterns confirmed that the observed hysteresis was associated with the transformation of **X-sql-1-Cu·CO**_**2**_ to **X-sql-1-Cu-B**. The second CO_2_ adsorption cycle revealed that the *in situ* obtained **X-sql-1-Cu-B** exhibited a type F-IV^16^ isotherm with a shifted gate-opening pressure (0.05 bar) compared to the first cycle. The *in situ* PXRD pattern at 1 bar for the second cycle is consistent with the same CO_2_-loaded phase of the first cycle, **X-sql-1-Cu·CO**_**2**_ as was the saturation uptake (133 cm^3^ g^–1^). The second cycle of desorption afforded **X-sql-1-Cu-B** (Figure S26). The third cycle of CO_2_ dosing involved the same phase transformations as those of the second cycle.

#### Temperature-Dependent Low-Pressure CO_2_ Cycling Experiment

**Figure 3:**
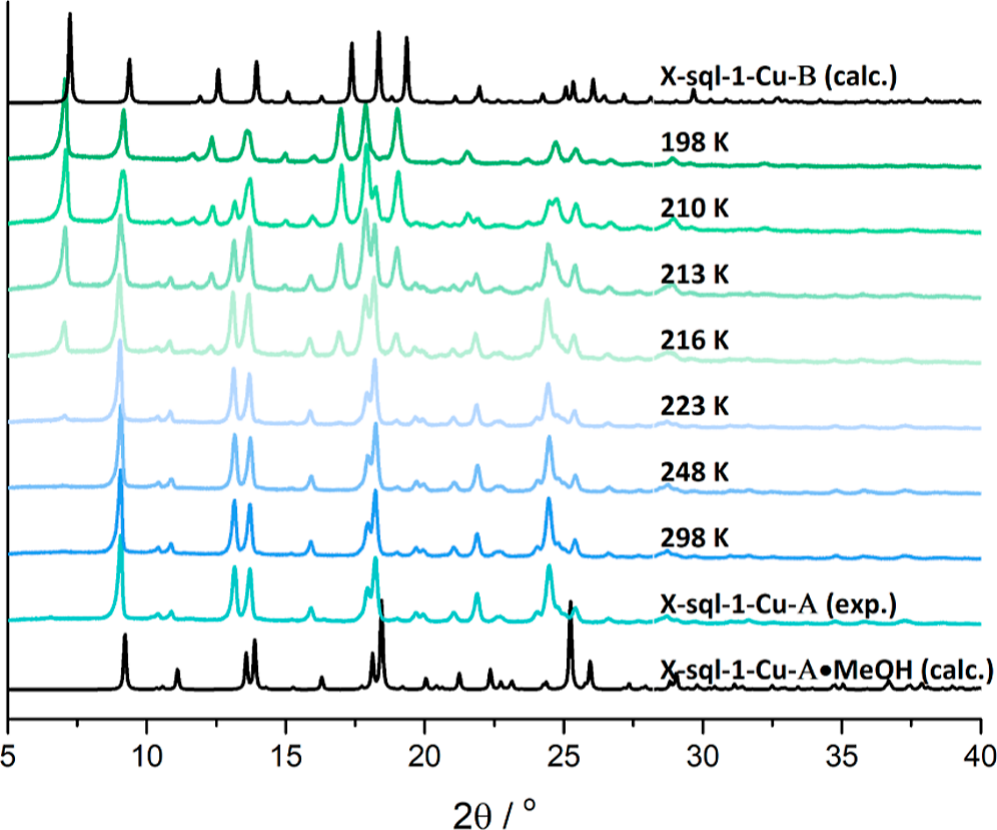
PXRD patterns of **X-sql-1-Cu** after 3 cycles of 1 bar CO_2_ exposure at temperatures from 198 to 298 K starting from **X-sql-1-Cu-A** in the first cycle.

Low-pressure sorption data collected for CO_2_ at 298 K and N_2_ at 77 K revealed negligible uptake (Figures S28 and S29), which prompted us to determine the effect of temperature on the transformation of **X-sql-1-Cu-A** to **X-sql-1-Cu-B**. A CO_2_ sorption-differential scanning calorimetry (DSC) experiment was thereby performed (see Supporting Information for experimental details). Activated **X-sql-1-Cu-A** was exposed to three cycles of 1 bar CO_2_ at temperatures ranging from 198 to 298 K (Figure S30), and the polymorphs were then monitored by conducting PXRD experiments after exposure. It was found that conversion of **X-sql-1-Cu-A** into **X-sql-1-Cu-B** occurred at 198 K, while for temperatures between 210 and 216 K, a mixture of the two polymorphs was formed (Figure 3). Samples exposed to CO_2_ between 223 and 298 K did not form **X-sql-1-Cu-B**, retaining the PXRD pattern of **X-sql-1-Cu-A**, indicating temperature dependence.

#### Low-Pressure C_2_H_2_ Sorption

**Figure 4:**
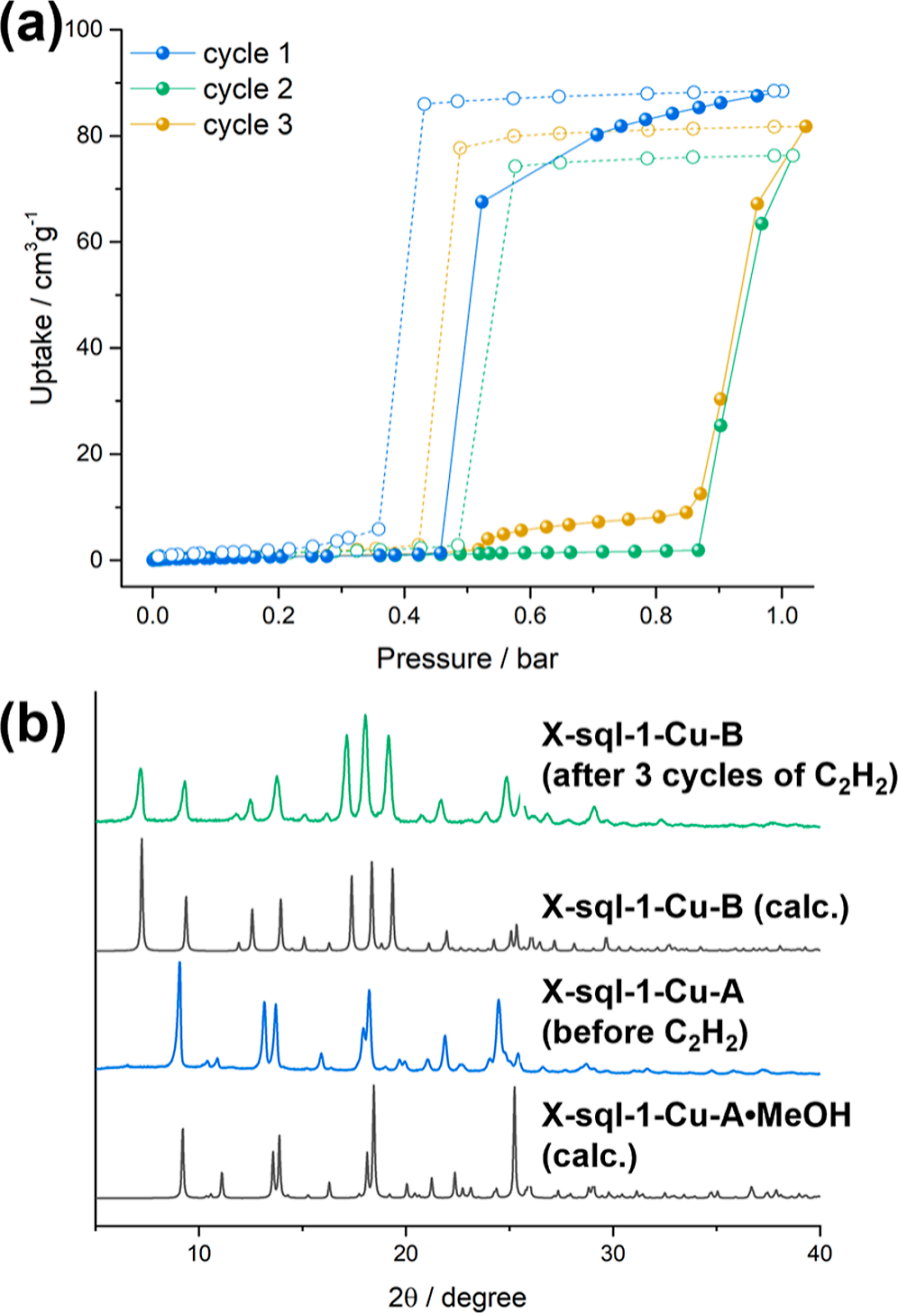
(a) Three cycles of C_2_H_2_ sorption/desorption starting with **X-sql-1-Cu-A** at 278 K and (b) PXRD patterns of **X-sql-1-Cu-A** measured before and after cycling sorption of C_2_H_2_.

Low-pressure C_2_H_2_ sorption isotherms were collected at 195, 273, 278, 288, and 298 K for **X-sql-1-Cu-B** (Figure S31). **X-sql-1-Cu-B** displayed a stepped type F-IV^16^ isotherm with gate-opening pressures of 0.04, 0.61, and 0.79 bar at 195, 273, and 278 K, respectively. To investigate the polymorphic transformation, a cycling experiment was performed on **X-sql-1-Cu-A** at 278 K, the highest temperature at which **X-sql-1-Cu-B** showed flexibility toward C_2_H_2_ (Figure 4a) below 1 bar. **X-sql-1-Cu-A** demonstrated a sharp type F-IV^16^ isotherm with an inflection point at *ca.* 0.5 bar and an uptake of 88.5 cm^3^ g^–1^ (or 3.95 mmol g^–1^) at 1 bar C_2_H_2_, with moderate hysteresis on desorption. The second consecutive cycle of C_2_H_2_ sorption revealed a shift of the inflection point to 0.87 bar and larger hysteresis on desorption when compared to the first cycle. The third cycle was found to be similar to the second one and PXRD revealed formation of **X-sql-1-Cu-B** after 3 cycles of C_2_H_2_ sorption (Figure 4b). These findings are consistent with the shifted gate-opening pressures seen in the second sorption cycle in **X-sql-1-Cu** induced by guests such as MeOH and CO_2_.

### Computational Studies

To explore the transformation of **X-sql-1-Cu-A** to **X-sql-1-Cu-B** during CO_2_ adsorption–desorption cycles (Scheme 1 and Figure 2), we performed density functional theory (DFT) calculations and grand canonical Monte Carlo (GCMC) simulations (see Supporting Information for details on the methodology). Construction and DFT optimization of open-pore forms of **-A** and **-B** were done based on the experimental unit cell parameters of the fully CO_2_-loaded phases **-A·CO**_**2**_ and **-B·CO**_**2**_ obtained from the *in situ* PXRD patterns (Figure S27). The open-phase structures were derived with the same crystal symmetry as their corresponding closed-phase structures.

**Figure 5:**
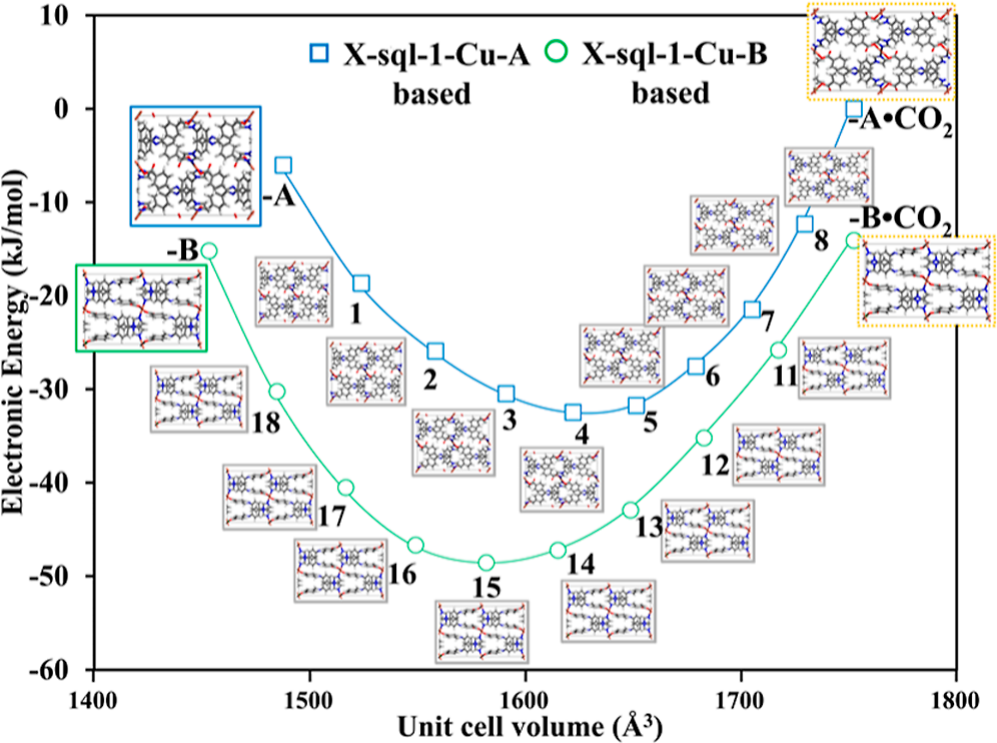
Empty host energy profiles for **X-sql-1-Cu-A**- and **X-sql-1-Cu-B**-derived structures as a function of unit cell volume.

Nudged elastic band simulations facilitated the construction of structures with intermediate unit cell volumes (*V*). Specifically, structures **1**–**8** were constructed between **X-sql-1-Cu-A** (*V* = 1486.13 Å^3^) and **-A·CO**_**2**_ (**9**, *V* = 1750.86 Å^3^), while structures **11**–**18** were constructed between **-B·CO**_**2**_ (**10**, *V* = 1750.86 Å^3^) and **X-sql-1-Cu-B** (*V* = 1451.71 Å^3^). In total, 18 CO_2_-loaded phases ranging from **X-sql-1-Cu-A** (**-A·CO**_**2**_**-1** to **-A·CO**_**2**_**-9**) and **X-sql-1-Cu-B** (**-B·CO**_**2**_**-10** to **-B·CO**_**2**_**-18**) underwent optimization through DFT calculations to determine their relative stability. The unit cell parameters of the optimized structures are presented in Table S6. Structures **-A·CO**_**2**_-**9** and **-B·CO**_**2**_-**10** share identical unit cell parameters, originating from **-A** and **-B** polymorphs, respectively. This is in agreement with experimental *in situ* PXRD analysis, which suggests identical CO_2_-loaded phases regardless of the starting polymorph (Figure 2b). DFT calculations provided insights into a crucial question regarding the behavior of this compound: why does **X-sql-1-Cu-A** transform to **X-sql-1-Cu-B** in response to CO_2_ adsorption–desorption? The answer lies in the lower energy barrier for CO_2_-loaded structures **X-sql-1-Cu-B·CO**_**2**_ (−14.1 kJ mol^–1^) in comparison to **X-sql-1-Cu-A·CO**_**2**_ (Figure 5).

**Figure 6:**
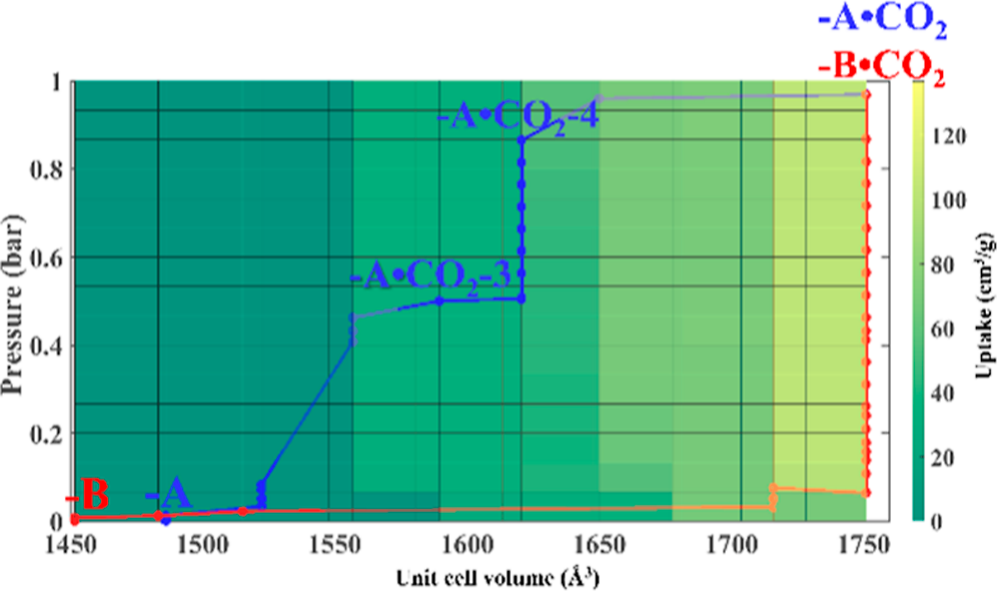
Contour plot representing GCMC-simulated CO_2_ adsorption isotherms for all structures derived from **X-sql-1-Cu-A** (blue) and **X-sql-1-Cu-B** (red).

The **X-sql-1-Cu-A** to **X-sql-1-Cu-B** transformation observed during an experimental adsorption–desorption CO_2_ process can be understood through GCMC simulations. Specifically, these simulations enabled us to visualize the CO_2_ uptake from simulated adsorption isotherms for structures at different unit cell volumes as a contour plot, onto which we can map the most plausible adsorption and desorption pathways (Figure 6). This analysis allowed us to track the position of the adsorbed CO_2_ in **X-sql-1-Cu-A**, noting intermediate pore openings ranging from structure **-A·CO**_**2**_**-3** (*V* = 1589.53 Å^3^) to **-A·CO**_**2**_**-4** (*V* = 1620.57 Å^3^) and, finally, **-A·CO**_**2**_**-9** (*V* = 1750.86 Å^3^) at full saturation (Figures S34–S36). These findings align with the experimental multistep isotherm observed during CO_2_ gas sorption on **X-sql-1-Cu-A** (Figure 2a). Conversely, the second adsorption cycle demonstrated a single-step isotherm for **X-sql-1-Cu-B**, reflecting minimal uptake until the instantaneous transformation to the open CO_2_-loaded phase, reaching maximum loading (Figures S37 and S38).

## Conclusions

In summary, we report a new CN with **sql** topology, **X-sql-1-Cu**, that crystallized as **X-sql-1-Cu-(MeOH)**_**2**_**·2MeOH** and readily transformed to two polymorphs, **X-sql-1-Cu-A** and **X-sql-1-Cu-B**, depending on applied conditions such as heat, N_2_ flow, or vacuum. **X-sql-1-Cu-A** and **X-sql-1-Cu-B** could be interconverted by sorption/desorption involving MeOH, CO_2_, or C_2_H_2_. Computational studies afforded energy landscapes for the two polymorphs, supporting that the CO_2_-loaded phase obtained from **X-sql-1-Cu-A** can transform to **X-sql-1-Cu-B** upon desorption, due to its lower energy. The two polymorphs exhibited differences in sorption behavior in terms of their threshold pressures for gate opening. This investigation highlights how polymorphism can be used to fine-tune sorption properties and is to our knowledge only the second such study involving **sql** networks.

## Supporting Information Available

Synthetic and characterization details: SCXRD, PXRD, TGA, DSC, NMR, SEM, gas sorption, and TOPOS analysis (PDF)
Crystallographic data for **X-sql-1-Cu-A·MeOH** (CIF)
Crystallographic data for **X-sql-1-Cu-B** (CIF)
Crystallographic data for **X-sql-1-Cu-(MeOH)**_**2**_**·2MeOH** (CIF)

## Supplementary Material

am4c03777_si_001.pdf

am4c03777_si_002.cif

am4c03777_si_003.cif

am4c03777_si_004.cif
