# Supplementary material for: Effect of Polymorphism on the Sorption Properties of a Flexible Square-Lattice Topology Coordination Network
Source: ACS Appl Mater Interfaces. 2024 Apr 26;16(18):24132–40. doi: 10.1021/acsami.4c03777 (PMC11082895; doi:10.1021/acsami.4c03777)
Supplement: Supplementary file 1 — am4c03777_si_001.pdf [file am4c03777_si_001.pdf]

Supporting information

Effect of polymorphism on the sorption properties of a flexible square-lattice topology coordination network

Aizhamal Subanbekova,[a] Andrey A. Bezrukov,[a] Volodymyr Bon,[b] Varvara I. Nikolayenko,[a] Kyriaki Koupepidou,[a] Debobroto Sensharma,[a] Sousa Javan Nikkhah, [a] Shi-Qiang Wang,[a,c] Stefan Kaskel,[b] Matthias Vandichel[a] and Michael J. Zaworotko[a]*

[a]Department of Chemical Sciences, Bernal Institute, University of Limerick, Limerick, V94 T9PX, Republic of Ireland.
E-mail: xtal@ul.ie
[b]Faculty of Chemistry, Technische Universitat Dresden, Bergstrasse 66, 01062 Dresden, Germany
[c]Institute of Materials Research and Engineering (IMRE), Agency for Science, Technology and Research (A*STAR), 2 Fusionopolis Way 138634, Singapore

Contents

1. **Polymorphism in sql metal-organic frameworks** 4

2. **Materials and synthesis** 11
   1. Materials 11
   2. Synthesis of ((4-(1H-Imidazol-1-yl)phenylimino)methyl)benzoic acid, HImibz 11
   3. Synthesis of [Cu(Imibz)$_2$·2MeOH], **X-sql-1-Cu-(MeOH)$_2$•2MeOH** 12
   4. Bulk synthesis of [Cu(Imibz)$_2$·2MeOH], **X-sql-1-Cu-(MeOH)$_2$•2MeOH** 12
   5. Preparation of **X-sql-1-Cu-A•MeOH** 12
   6. Preparation of **X-sql-1-Cu-B** 12

3. **Characterization** 13
   1. Single-Crystal X-ray Diffraction (SCXRD) 13
   2. Thermogravimetric Analysis (TGA) 18
   3. SC XRD comparison 19
   5. Powder X-ray Diffraction (PXRD) 24
   6. Variable Temperature Powder X-ray Diffraction (VTPXRD) 26

4. **Polymorphs interconvertibility** 29
   1. MeOH sorption studies 29
   2. Scanning Electron microscopy (SEM) 29
   3. Water vapor sorption 34
   4. Low Pressure Gas sorption 36
   5. *In situ* Differential Scanning Calorimetry (DSC) 40
   6. *In situ* Powder X-ray Diffraction 41

5. Computational methodology 43

1. Density Functional Theory calculations 43

2. Grand Canonical Monte Carlo simulations 45

6. References 53

1. Polymorphism in sql metal-organic frameworks

Polymorphism identification using database mining.

The list of MOFs having **sql** net topology was obtained from the TTO TOPOS database (version: December 2021);[1] valence-bonded MOFs in standard representation were used (9687 crystal structures). The MOF crystal structures from the TTO database were analyzed using queries to the Cambridge Structural Database (CSD version 5.43+3 updates, September 2022) through the CSD Application Programming Interface.[2] The datamining strategy used in this work to identify polymorph groups of **sql** networks schematically illustrated in Figure S1

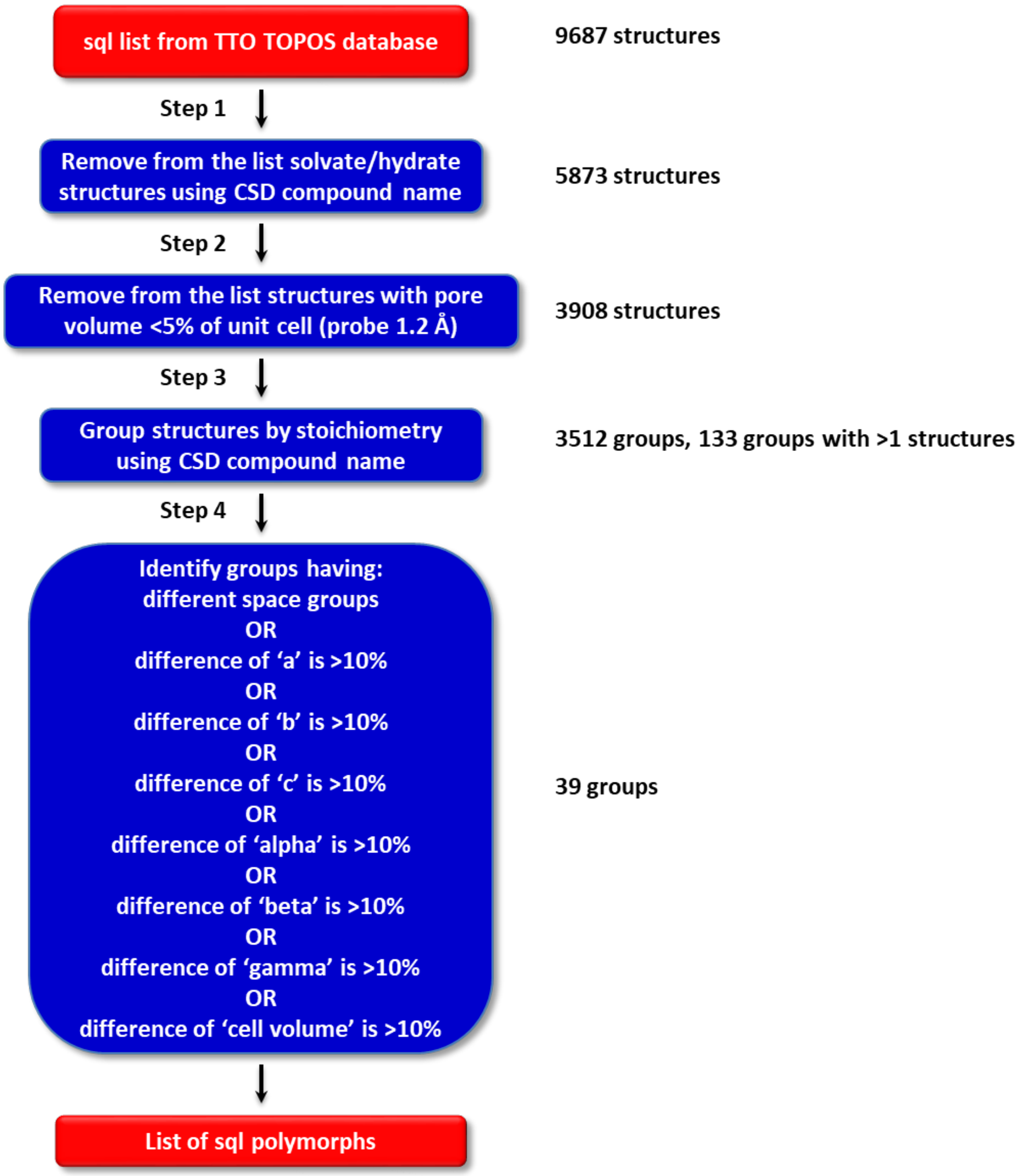

Figure S 1. Flow chart of the algorithm used for identification of **sql** network polymorphs.

Table S 1*. List of polymorphs in* ***sql*** *MOFs.*

Polymorph example #	Formula	Linker	RefCode	Reference
**1**	$(C_{10} H_8 Cl_2 N_2 Pb)_n$	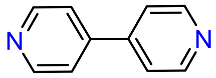	ABIXAL	[3]
			ABIXAL01	[4]
			ABIXAL02	
			ABIXAL03	
**2**	$(C_{10} H_8 F_3 Mn N_2)_n$	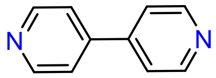	WUXMOR	[5]
			WUXMOR01	
			WUXMOR02	
			WUXMOR03	
**3**	$(C_{10} H_8 Cl_2 Cu N_2)_n$	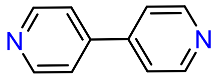	TISBAY	[6]
			TISBAY01	[7]
**4**	$(C_{10} H_8 Cd I_2 N_2)_n$	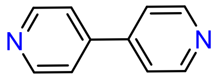	IPECEL01	[8]
			IPECEL06	
			IPECEL07	[9]
			IPECEL08	CSD Communication
**5**	$(C_{10} H_8 Cd Cl_2 N_2)_n$	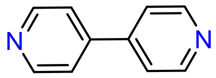	IPEBIO	[8]
			IPEBIO01	
**6**	$(C_{52} H_{42} Fe_2 N_4 O_6 S_2 Zn)_n$	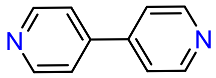	QIZLAN	[10]
			QOFNEF	

**7**	$(C_{10}H_8CdCl_2N_2)_n$	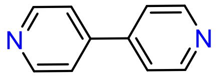	IPEBIO02	11
			IPEBIO03	12
**8**	$(C_4H_4CdCl_2N_2)_n$	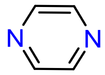	TISSUJ	13
			TISSUJ02	14
**9**	$(C_8H_8Cl_2CuN_4O_8)_n$	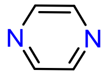	CUPYZP01	15
			CUPYZP02	
**10**	$(C_{12}H_8FeN_8Ni)_n$	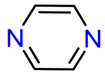	EWAKAP	16
			EWAKAP01	
			EWAKIX	
			EWAKIX01	
**11**	$(C_{12}H_8CoN_8Ni)_n$	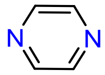	GUSNUE	17
			GUSNUE01	
**12**	$(C_4H_6O_4Zn)_n$	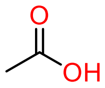	ZNACEU01	18
			ZNACEU04	19
**13**	$(C_4H_4O_{10}Th)_n$	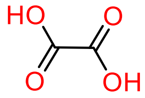	ZZZTRQ01	20
			ZZZTRQ02	
			ZZZTRQ03	
**14**	$(C_{22}H_{22}N_4O_4Zn)_n$	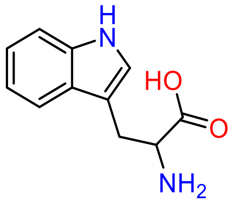	HUHCOD	21
			HUHCOD01	22
**15**	$(C_{22}H_{18}CdO_6)_n$	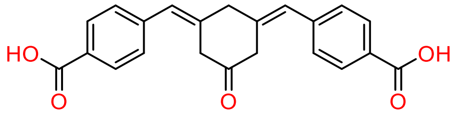	HUMYUM	23
			HUMYUM01	
**16**	$(C_4N_6Zn)_n$		HAXFIV	24

		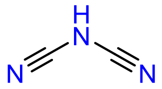	HAXFIV01	
			HAXFIV02	
**17**	$(C_{10} H_{11} Cd N_7 Ni)_n$	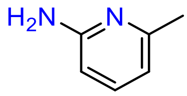	DIMYAZ01	[25]
			DIMYAZ20	[26]
**18**	$(C_{28} H_{24} Cu F_4 N_{14} O_2 S_2)_n$	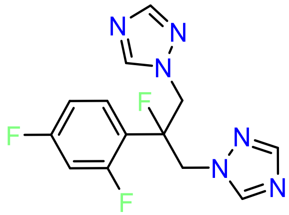	PIBKUH	[27]
			PIBLAO	
**19**	$(C_{11} H_9 Mn O_6 P)_n$	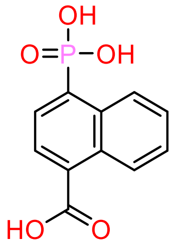	PULFOU	[28]
			PULFOU01	
**20**	$(C_8 H_{14} Cd O_2 S_4)_n$	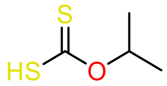	CDIPXT	[29]
			CDIPXT01	[30]
**21**	$(C_4 H_8 N_2 Ni O_6)_n$	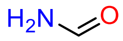	NIXQAO	[31]
			NIXQAO01	
**22**	$(C_{12} H_{12} Mn N_8)_n$	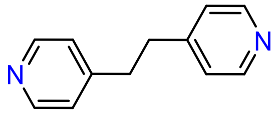	LAXKUQ	[32]
			LAXKUQ01	[33]
**23**	$(C H_5 Fe O_4 P)_n$	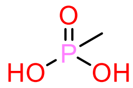	UFAQUN	[34]
			UFAQUN02	[35]
**24**	$(C_{15} H_{15} Cu N_3 O_4)_n$	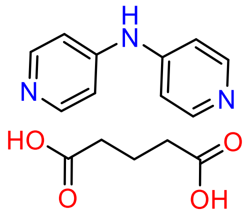	HOQBAR	[36]
			HOQBAR01	
**25**	$(C_{12} H_{26} Cd_2 N_{10} S_4)_n$		AEATCD01	[37]

		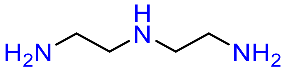	AEATCD10	38
**26**	$(C_6 H_{10} O_4 Zn)_n$	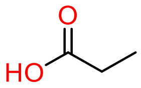	ZNPROP	39
			ZNPROP01	7
**27**	$(C_8 H_8 Cd_2 N_8 O_{12})_n$	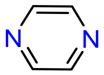	CECRUY	11
			CECRUY01	
**28**	$(C_{33} H_{22} Co N_4 O_7)_n$	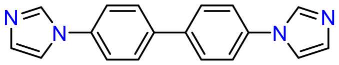 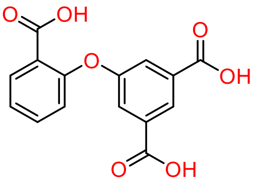	TAXTUK	40
**29**			TAXVAS	
**30**	$(C_{14} H_8 Cl_2 Fe N_6 Pt)_n$	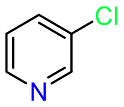	MUHNAF	41
			MUHNAF01	
			MUHNAF02	
**31**	$(C_{12} H_{12} Cd N_2 O_6)_n$	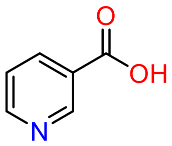	WIHWUG	42
			WIHWUG01	43
**32**	$(C_{20} H_{26} F_6 N_4 Ni O_4)_n$	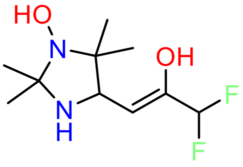	POFKIE	44
			POFKIE01	
**33**	$(C_{22} H_{26} N_4 O_8 Zn)_n$	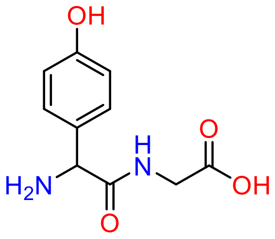	QEYZAY	45
			QEYZAY01	
			QEYZAY02	
			QEYZAY03	
			QEYZAY04	
			QEYZAY05	

			QEYZAY06	
**34**	$(C_{22} H_{14} Fe N_6 Pd)_n$	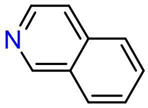	SUQFIW	[46]
			SUQFIW01	
**35**	$(C_{28} H_{16} Au_4 F_4 Fe_2 N_{12})_n$	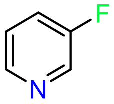	NIXVOG02	[47]
			NIXVOG03	[48]
**36**	$(C_{20} H_{18} N_4 Ni O_6 S_2)_n$	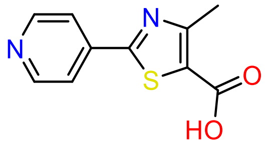	JEVBOC	[49]
			JEVBOC01	
**37**	$(C_8 H_6 Cd O_5)_n$	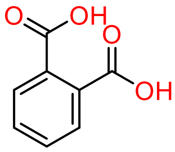	KACBOF	[50]
			KACBOF01	[51]
			KACBOF02	[52]

2. Materials and synthesis

1. Materials

All reagents and solvents were procured from Sigma Aldrich and used without further purification.

2. Synthesis of ((4-(1H-Imidazol-1-yl)phenylimino)methyl)benzoic acid, HImibz

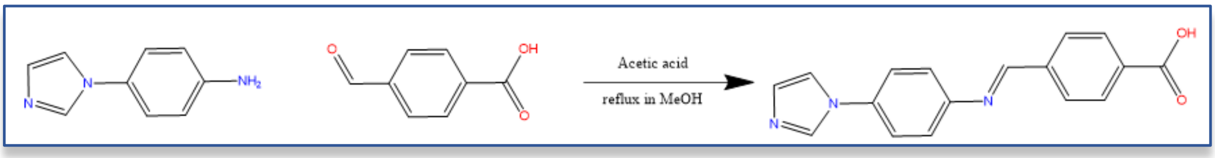

The ligand HImibz ((4-(1H-Imidazol-1-yl)phenylimino)methyl)benzoic acid was synthesised following modified literature method.[53] 4-(1H-imidazol-1-yl)aniline (4700 mg, 30 mmol), 4-carboxybenzaldehyde (3000 mg, 20 mmol) and acetic acid (5 mL) and MeOH (86 mL) were combined in a 250 mL round-bottom flask. The color of the solution changed from colorless into pale yellow. The reaction was refluxed overnight, washed with MeOH and dried in air (88.7 % yield, ^{1}H NMR (270 MHz, DMSO-d_6) δ 7.1, 7.45, 7.7, 8.05, 8.3, 8.75.

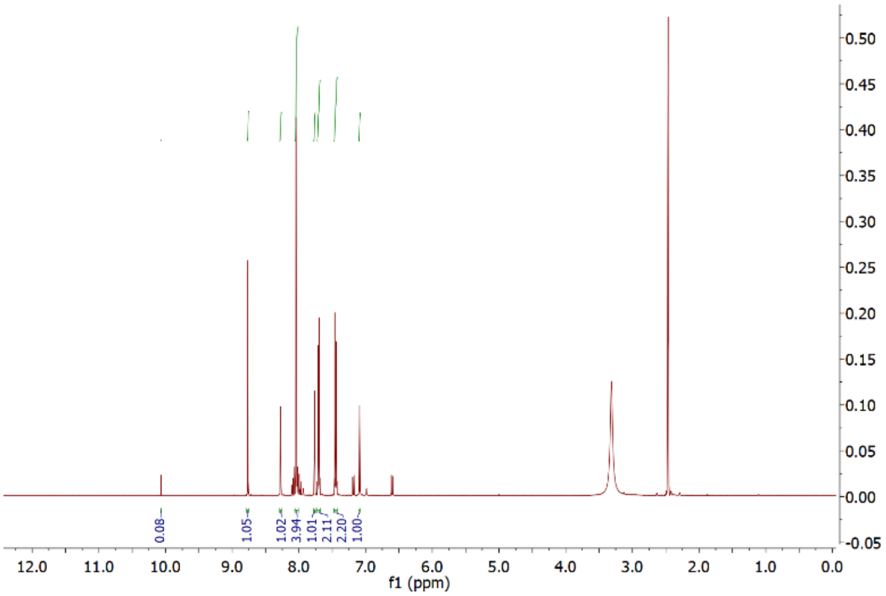

Figure. S 2. NMR of HImibz linker in d_6-DMSO.

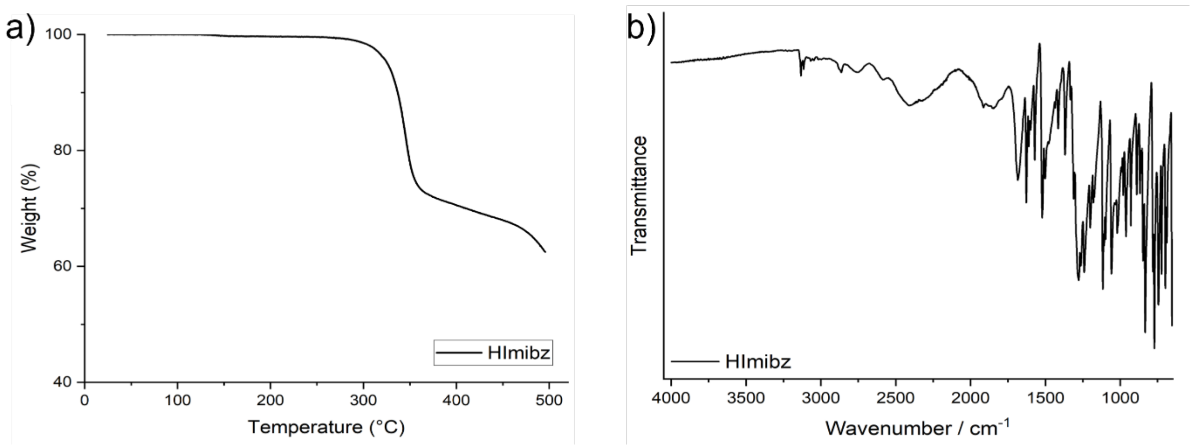

Figure. S 3. a) TGA and b) FTIR of HImibz.

3. Synthesis of [Cu(Imibz)$_2$·2MeOH], **X-sql-1-Cu-(MeOH)$_2$•2MeOH**

A solution of HImibz (10 mg, 0.03 mmol) in 2 mL of DMF was added to test tube, 2 mL of DMF:MeOH (1:1) was carefully layered over the solution to act as a buffer layer. Finally, a solution of Cu(NO$_3$)$_2$·3H$_2$O (12 mg, 0.05 mmol) in 2 mL MeOH was layered over this buffer layer, and the tube was left undisturbed for 2 weeks. Blue needle crystals of Cu(Imibz)$_2$·2MeOH (as-synthesized **X-sql-1-Cu-(MeOH)$_2$•2MeOH** were obtained.

4. Bulk synthesis of [Cu(Imibz)$_2$·2MeOH], **X-sql-1-Cu-(MeOH)$_2$•2MeOH**

A solution of Cu(NO$_3$)$_2$·3H$_2$O (145.5 mg, 0.5 mmol) in 2.5 mL MeOH was added to a solution of HImibz (60.25 mg, 0.25 mmol) in 5 mL of DMF and heated in the oven at 60 °C overnight. A blue crystals of as-synthesized **X-sql-1-Cu-(MeOH)$_2$•2MeOH** were obtained.

5. Preparation of **X-sql-1-Cu-A•MeOH**

X-sql-1-Cu-A•MeOH was obtained by drying as-synthesized **X-sql-1-Cu-(MeOH)$_2$•2MeOH** on air for 20 mins.

6. Preparation of **X-sql-1-Cu-B**

X-sql-1-Cu-B was isolated by exposing as-synthesized **X-sql-1-Cu-(MeOH)$_2$•2MeOH** to vacuum and heating. Alternatively, **X-sql-1-Cu-B** was obtained by resolvation of **X-sql-1-Cu-A•MeOH** in MeOH and under N_2 flow.

3. Characterization

1. Single-Crystal X-ray Diffraction (SCXRD)

Single crystal X-ray diffraction data for **X-sql-1-Cu-(MeOH)$_2$•2MeOH** was collected on a Bruker Quest diffractometer equipped with a CMOS detector and IμS microfocus X-ray source (Cu Kα, λ = 1.54178 Å) under N_2 flow at 100 K. SCXRD data for **X-sql-1-Cu-A•MeOH** and **X-sql-1-Cu-B** was collected using the same procedure.

All SCXRD data were indexed, integrated, and scaled in APEX4.[54] Absorption correction was performed by multi-scan method using SADABS.[55] Space group determination was achieved using XPREP4 implemented in APEX4. Structures were solved using intrinsic phasing method (SHELXT) and refined on F^2 using nonlinear least-squares techniques with SHELXL[56] programs incorporated in X-seed graphical user interface. Anisotropic thermal parameters were applied to all non-hydrogen atoms. Hydrogen atoms were placed in calculated positions using riding models. SCXRD data of **X-sql-1-Cu-(MeOH)$_2$•2MeOH** revealed a non-interpenetrated square lattice structure. In X-sql-1-Cu-B, half of Imibz ligand and aromatic ring are disordered over two positions. The voids space of all the crystal structures was calculated by using PLATON.[57]

Table S 2. *Single crystal X-ray data for all three phases of X-**sql**-1-Cu.*

	**X-sql-1-Cu-(MeOH)$_2$•2MeOH**	**X-sql-1-Cu-A•MeOH**	**X-sql-1-Cu-B**	**X-sql-1-Cu-C•CO2**
CCDC	2321908	2321906	2321907	
Method	SC	SC	SC	powder
Formula	$C_{34}H_{24}N_6O_4Cu$ + solvent	$C_{34}H_{24}N_6O_4Cu$ + MeOH	$C_{34}H_{24}N_6O_4Cu$	
Formula weight (g/mol)	706.21	770.98	665.07	

Temperature (K)	100	100	100	195
Wavelength (Å)	1.54056	1.54056	1.54056	1.54056
Crystal system	Monoclinic	Monoclinic	Monoclinic	Monoclinic
Space group	*P21/c*	*P21/c*	*P21/c*	*P21/c*
a (Å)	13.7187(8)	9.6790(3)	12.2390(5)	13.227(6)
b (Å)	18.7998(11)	17.0590(4)	14.8570(4)	15.333(15)
c (Å)	7.5113(4)	9.0825(3)	8.0244(3)	8.817(3)
α (°)	90	90	90	90
β (°)	103.543(2)	97.9700	95.775(2)	101.74(15)
γ (°)	90	90	90	90
V ($Å^3$)	1883.37(19)	1486.13(8)	1451.71(9)	1750.6(3)
Z	2	2	2	
D_c (g/cm^3)	1.245	1.524	1.515	
R_1 [$I > 2\sigma(I)$]	0.0487	0.0454	0.0600	
WR_2 [all data]	0.1339	0.1272	0.1633	
Rwp				10.14%

The CheakCIF **X-sql-1-Cu-B** contains the following alerts:

Alert level B

PLAT088_ALERT_3_B Poor Data / Parameter Ratio .... 7.68 Note

Response: Single crystals of **X-sql-1-Cu-B** were collected by removing coordinated and guest methanol molecules of the as-synthesised phase. This results in a structural transformation that significantly impacted the crystal quality. We therefore attribute the resulting B alert to poor diffraction. Moreover, the entire molecule was found disordered over two positions. Several restraints (RIGU and SIMU) were used to improve refinement stability.

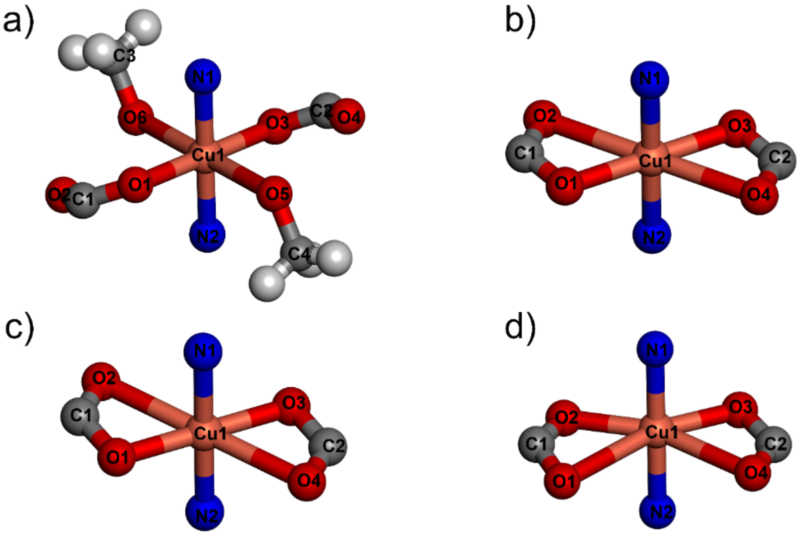

Figure S 4. *Coordination environment around Cu center: a)* ***X-sql-1-Cu-(MeOH)$_2$•2MeOH****, b)* ***X-sql-1-Cu-A•MeOH****, c)* ***X-sql-1-Cu-B*** *(disorder 1) and d)* ***X-sql-1-Cu-****B (disorder 2).*

Table S 3*. List of angles around Cu center in* ***X-sql-1-Cu****.*

**Angles**	**X-sql-1-Cu-(MeOH)$_2$•2MeOH**	**X-sql-1-Cu-A•MeOH**	**X-sql-1-Cu-B (disorder 1)**	**X-sql-1-Cu-B (disorder 2)**
**∠N1-Cu1-N2**	180 °	180 °	180 °	180 °
**∠O1-Cu1-N1**	88.9 °	90.5 °	98.2 °	90.7 °
**∠O1-Cu1-N2**	91.1 °	89.6 °	81.8 °	89.3 °
**∠O6-Cu1-N1**	85.44 °	-	-	-
**∠O6-Cu1-N2**	94.6 °			
**∠O2-Cu1-N1**	-	88.1 °	89.5 °	82.9 °
**∠O2-Cu1-N2**		91.9 °	90.5 °	97.1 °
**∠O1-Cu1-O3**	180 °	180 °	180 °	160.6 °
**∠O1-Cu1-O4**	-	123.3 °	121.7 °	140.1 °

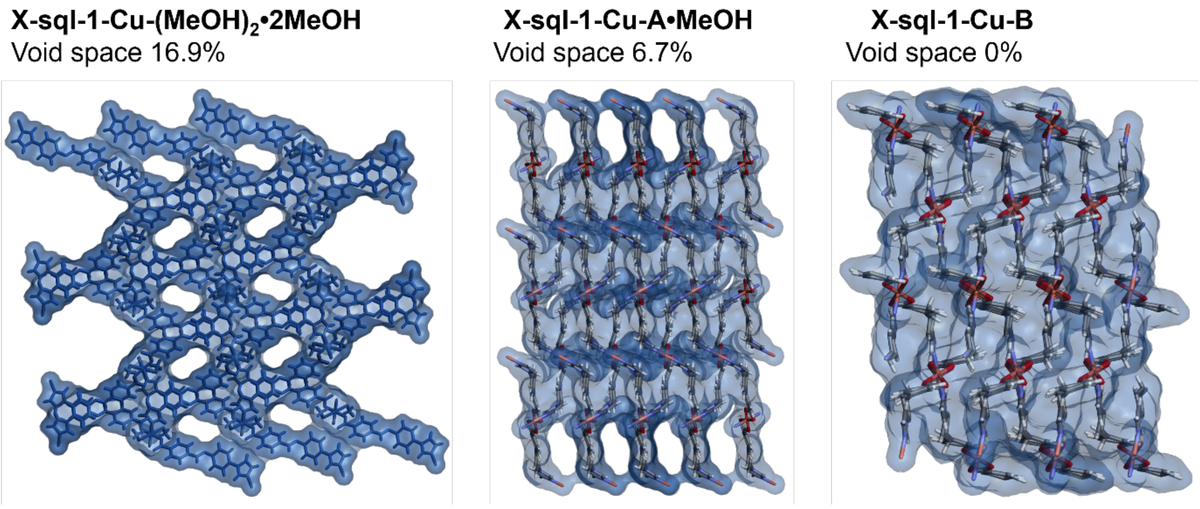

Figure S 5. Connolly map of **X-sql-1-Cu-(MeOH)$_2$•2MeOH**, **X-sql-1-Cu-A•MeOH** and **X-sql-1-Cu-B**.

2. Thermogravimetric Analysis (TGA)

Thermogravimetric analysis (TGA) was conducted using a TA Instruments Q50 system. Each sample was loaded into an aluminium pan and heated at 10 K min-1 from room temperature to 773 K under a continuous flow of N_2.

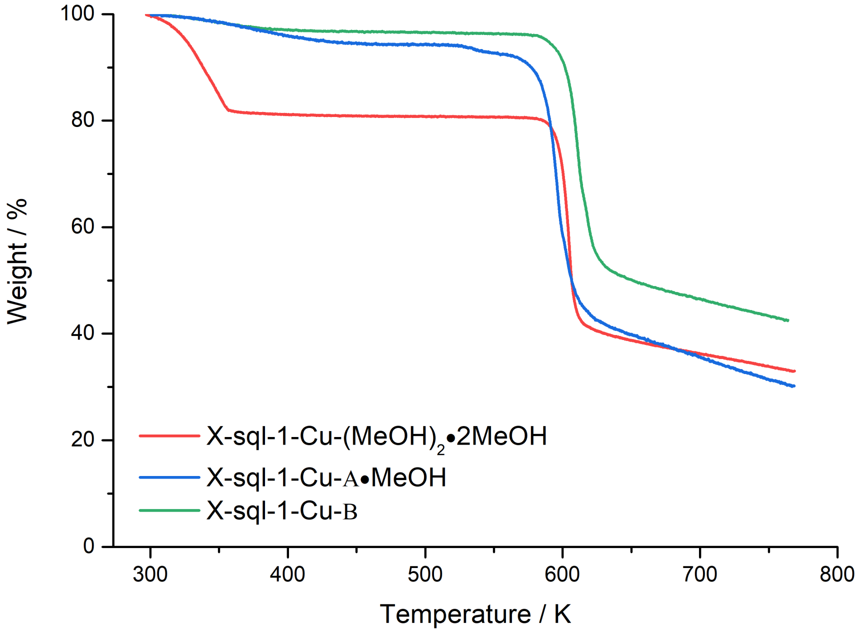

Figure. S 6. Thermogram of **X-sql-1-Cu** under continuous N_2 flow.

3. SC XRD comparison

Comparison of the SCXRD structures of three phases of **X-sql-1-Cu** revealed that the coordination environment of the Cu centers had changed, with differences in the N-Cu-C_{COO-} angles: 81.29°, 92.28° and 95.93° for **X-sql-1-Cu-(MeOH)$_2$•2MeOH**, **X-sql-1-Cu-A•MeOH** and **X-sql-1-Cu-B**, respectively (Figure S4 and Table S3). The Imibz coordination mode underwent transformation from monodentate to chelating and the internetwork Cu···Cu distance decreased from 18.20 Å in **X-sql-1-Cu-(MeOH)$_2$•2MeOH** to 17.80 Å and 17.89 Å in **X-sql-1-Cu-A•MeOH** and **X-sql-1-Cu-B**, respectively (Figure S8). Rotation and bending of the imidazole and phenyl rings with respect to the central ring of Imibz led to shortening of the diagonal Cu···Cu distance within the **sql** net from 18.80 Å in **X-sql-1-Cu-(MeOH)$_2$•2MeOH** to 17.06 Å and 14.86 Å in **X-sql-1-Cu-A•MeOH** and **X-sql-1-Cu-B**, respectively (Figure S8).

Comparison of the single crystal structures of **X-sql-1-Cu-A•MeOH** and **X-sql-1-Cu-B** revealed that the dihedral angle formed by the benzoate and central phenyl ring of Imibz was 67.21° in **X-sql-1-Cu-A•MeOH** *vs.* 8.73° and 13.27° in the two disordered components of **X-sql-1-Cu-B** (Figure S9). The second dihedral angle formed by the central ring and imidazole ring decreased from 27.20° to 70.89° and 82.51° in **X-sql-1-Cu-A•MeOH** and **X-sql-1-Cu-B**, respectively (Figure S9). **X-sql-1-Cu-B** exhibited a shorter interlayer distance than **X-sql-1-Cu-(MeOH)$_2$•2MeOH** and **X-sql-1-Cu-A•MeOH** (8.44 vs. 10.12 Å and 9.66 Å, respectively), resulting in **X-sql-1-Cu-B** being non-porous (Figure 1d). Furthermore, the torsion of Imibz linker resulted in a reduction in interlayer distances in **X-sql-1-Cu-B** indicating it's denser compared to **X-sql-1-Cu-A•MeOH** (Figure 10). Table S4 demonstrates the detailed comparison of these interlayer distances.

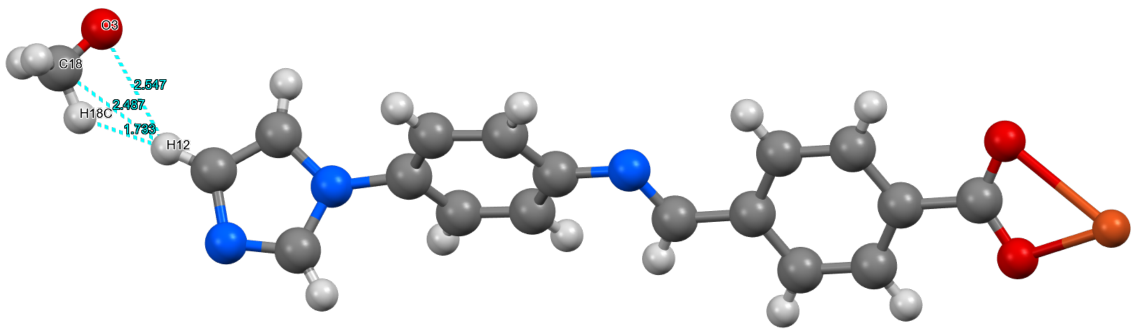

Figure S 7. **X-sql-1-Cu-A•MeOH** polymorph and short contacts distances with adjacent MeOH molecule.

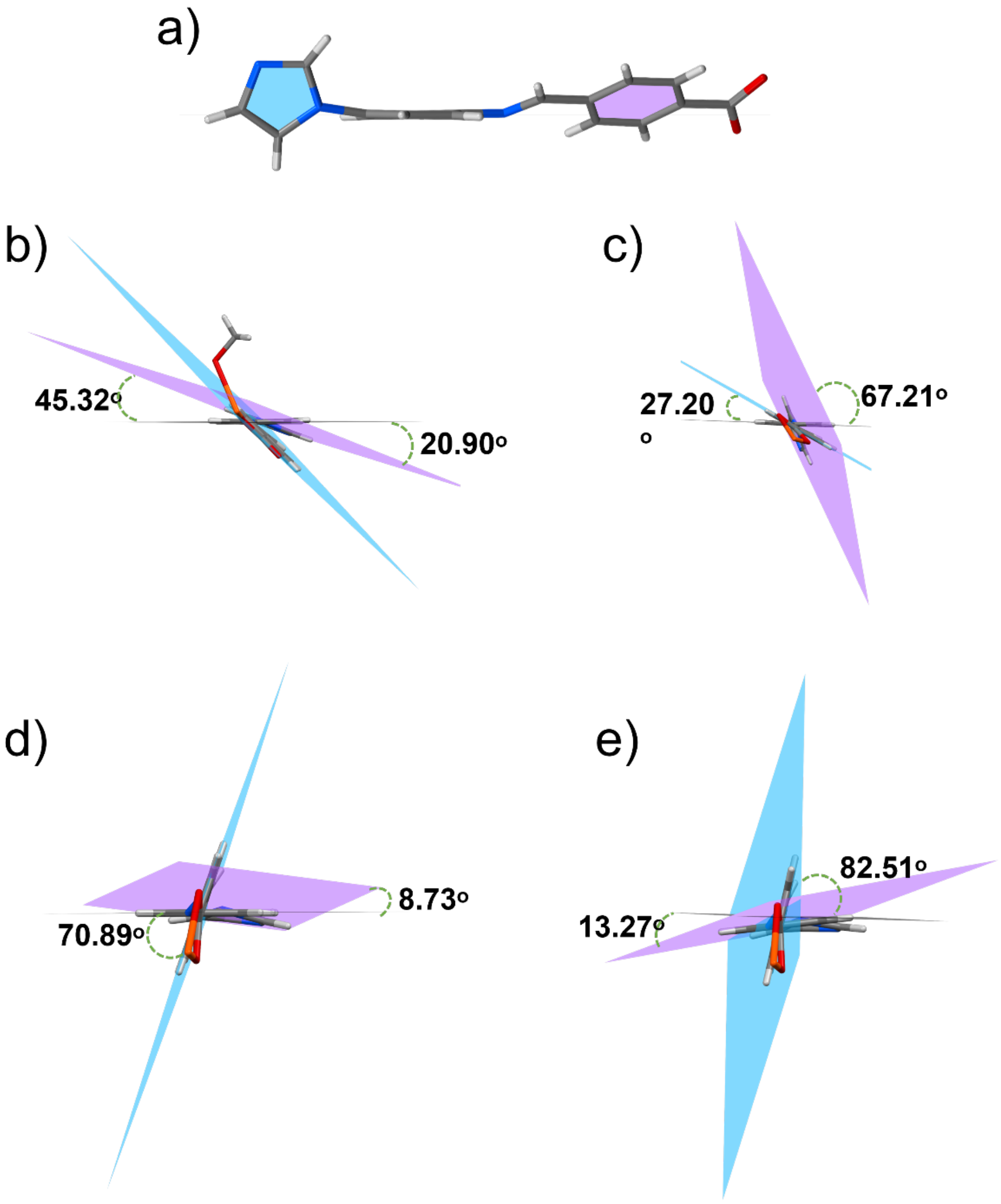

Figure S 8. Angles between 3 planes, corresponding to aromatic rings of a) **X-sql-1-Cu-(MeOH)$_2$•2MeOH**, b) **X-sql-1-Cu-A•MeOH** c) **X-sql-1-Cu-B** (disorder 1) and d) **X-sql-1-Cu-B** (disorder 2).

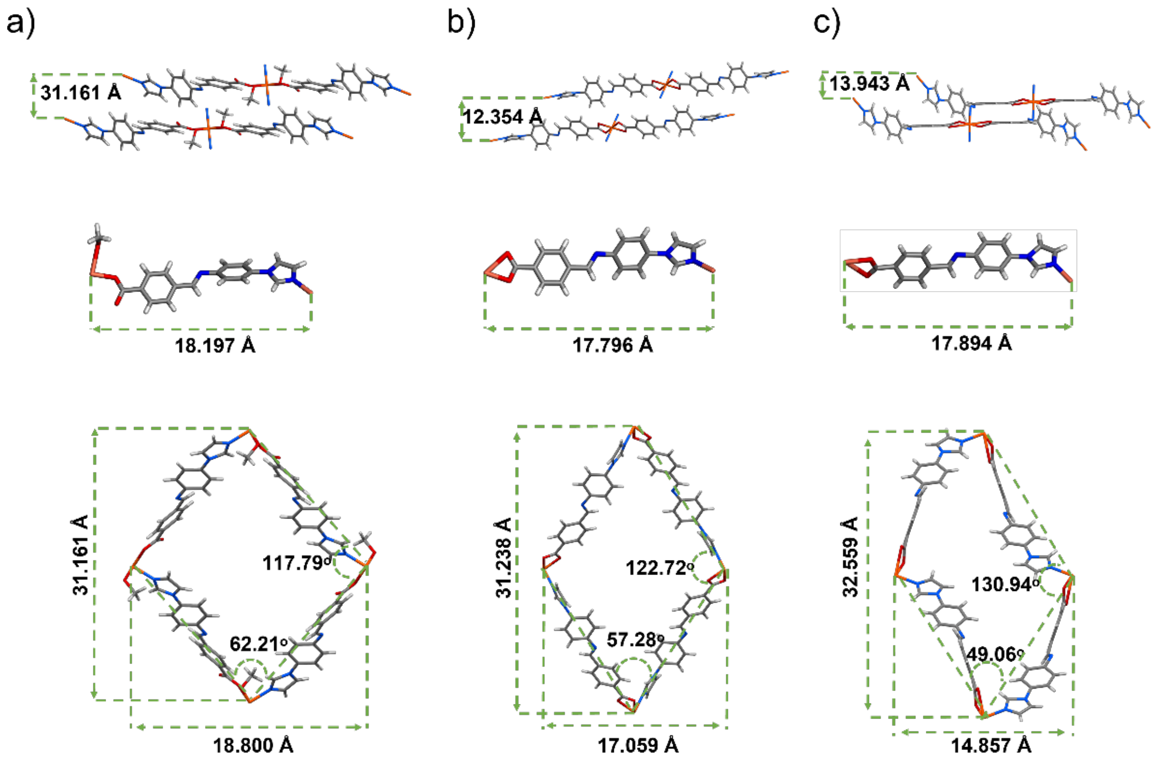

Figure S 9. Angles and distances in all three polymorphs of **X-sql-1-Cu**: a) **X-sql-1-Cu-(MeOH)$_2$•2MeOH**, b) **X-sql-1-Cu-A•MeOH** and c) **X-sql-1-Cu-B.**

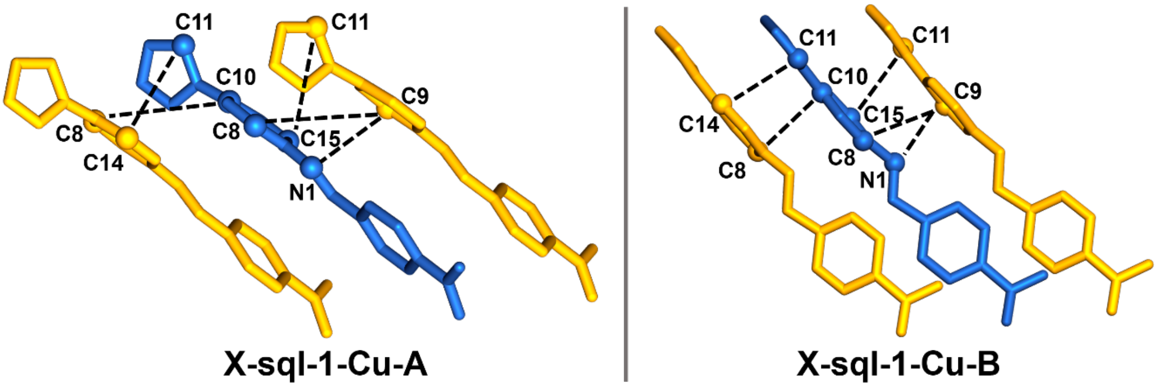

Figure S 10. Interlayer distances in **X-sql-1-Cu-A•MeOH** and **X-sql-1-Cu-B**.

Table S 4. *List of distances between layers in* ***X-sql-1-Cu****.*

**Distances**	**X-sql-1-Cu-A•MeOH / Å**	**X-sql-1-Cu-B / Å**
C11···C14	5.8	3.4
C10···C8	4.0	3.3
C8···C9	3.8	3.4
C15···C11	5.5	3.3
N1···C9	3.3	3.2

5. Powder X-ray Diffraction (PXRD)

Powder X-ray diffraction (PXRD) experiments were conducted using microcrystalline samples on a PANalytical Empyrean diffractometer (40 kV, 40 mA, Cu $K_{\alpha1,2,}$ λ = 1.5418 Å) in Bragg Brentano geometry. Data was collected from 5 to 40˚ 2θ with a step size of 0.0262606˚ and a scan time of 55 seconds per step. Powder samples were evenly distributed on a zero-background Si sample holder. Data analysis was carried out using X'Pert HighScore Plus8 (Version 2.2e).[58] Reference powder patterns were calculated from SCXRD structures using Mercury.[59]

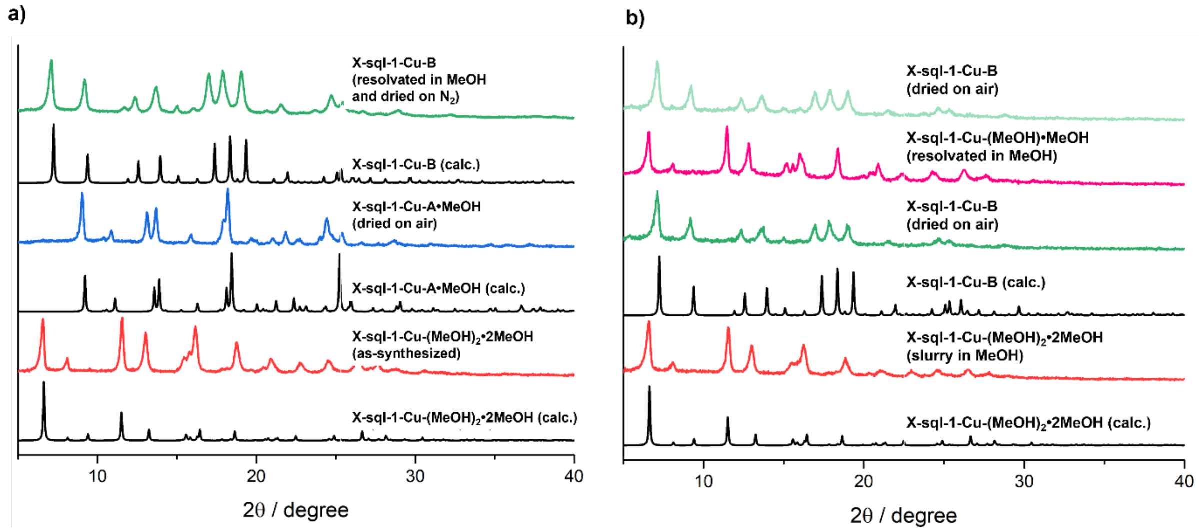

Figure S 11. PXRD patterns confirm the phase transformation of a) solvothermal synthesis in DMF:MeOH and b) of slurry in MeOH.

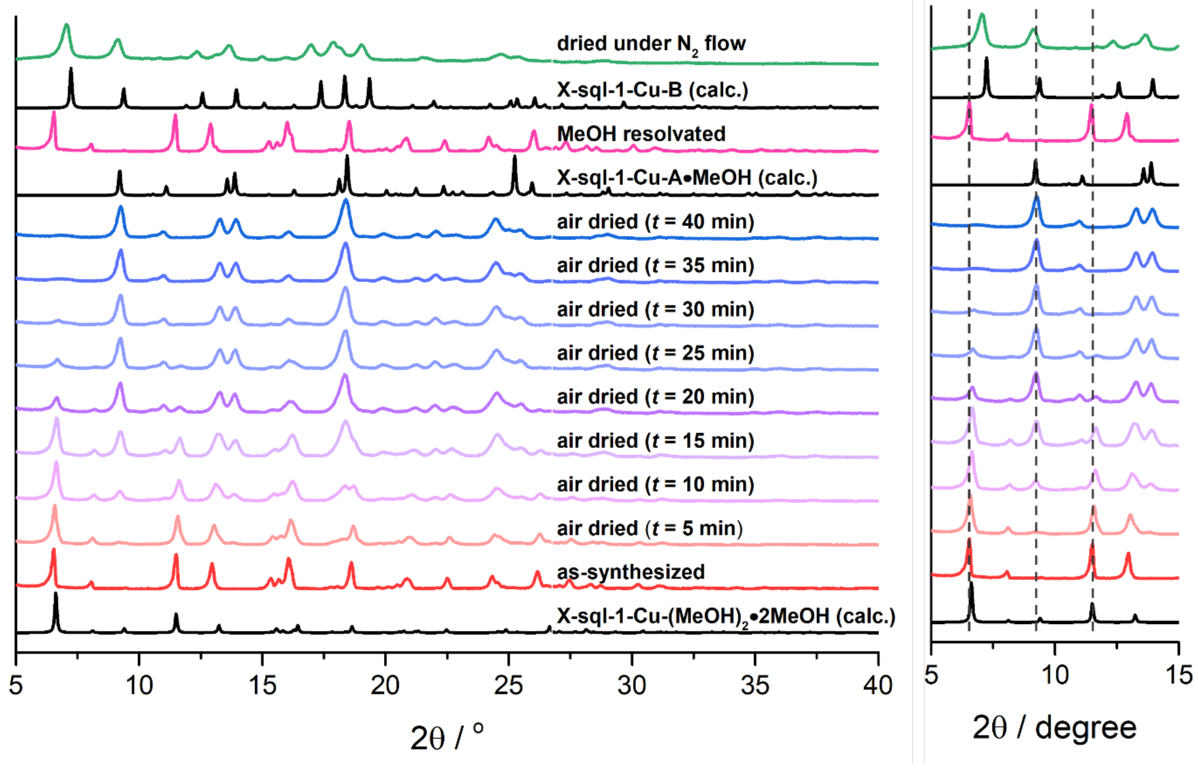

Figure S 12. PXRD pattern of MeOH solvation/desolvation cycling starting from **X-sql-1-Cu-(MeOH)$_2$•2MeOH**.

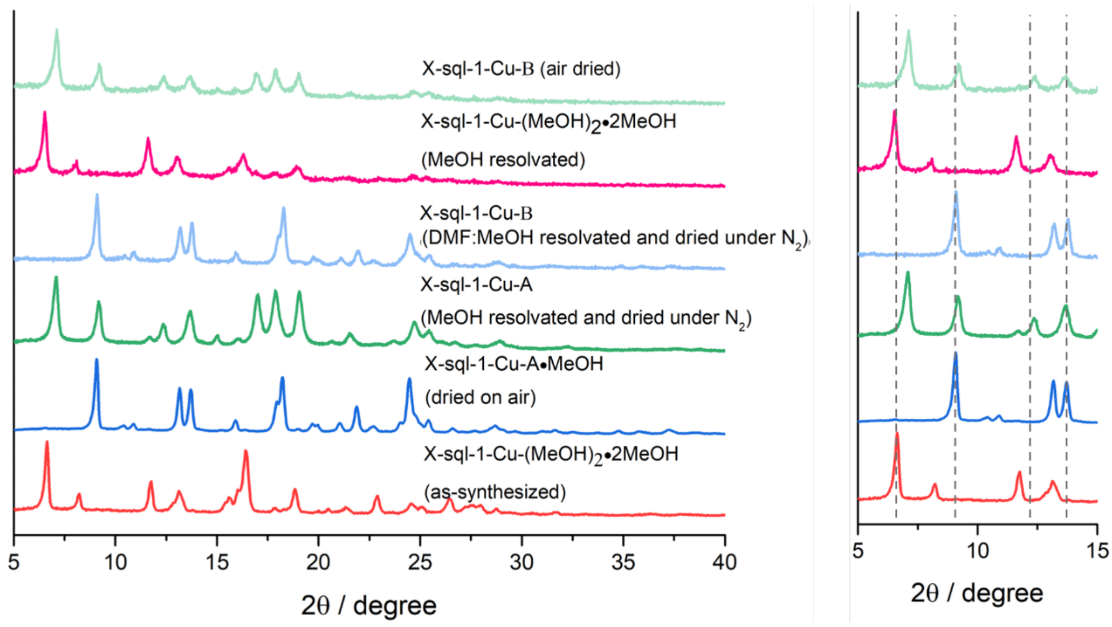

Figure S 13. PXRD patters of phase transformations starting from as-synthesized **X-sql-1-Cu-(MeOH)$_2$•2MeOH** and resolvating it in DMF and MeOH.

6. Variable Temperature Powder X-ray Diffraction (VTPXRD)

Diffractograms at different temperatures were recorded using a PANalytical X'Pert Pro-MPD diffractometer equipped with a PIXcel3D detector operating in scanning line detector mode. Anton Paar TTK 450 stage coupled with the Anton Paar TCU 110 Temperature Control Unit was used to record the variable temperature diffractograms. The diffractometer is outfitted with an Empyrean Cu LFF (long fine focus) HR (9430 033 7300x) tube operated at 40 kV and 40 mA and CuKα radiation (λ_α = 1.54056 Å) was used for diffraction experiments. Continuous scanning mode with the goniometer in the theta-theta orientation was used to collect the data. Incident beam optics included the Fixed Divergences slit, with a 1/4° divergence slit and a Soller slit (0.04 rad). Divergent beam optics included a P7.5 S7 anti-scatter slit, a Soller slit (0.04 rad), and a Ni-β filter. In a typical experiment, 50 mg of sample was ground into a fine powder and was loaded on a zero-background disc made for Anton Paar TTK 450 chamber. The data was collected from 4°-40° (2θ) with a step-size of 0.0167113° and a scan time of 200 seconds per step. Crude data were analyzed using the X'Pert HighScore Plus[9]™ software V 4.1 (PANalytical, The Netherlands).

VT-PXRD (298 - 473 K) experiments under air, continuous nitrogen flow and dynamic vacuum were performed to study the thermal stability and interconversion of the **X-sql-1-Cu** polymorphs. Exposure of as-synthesized **X-sql-1-Cu-(MeOH)$_2$•2MeOH** to a continuous nitrogen flow and heating triggered transformation to **X-sql-1-Cu-A** at 313 K (Figure S12). With additional heating up to 473 K the PXRD of obtained **X-sql-1-Cu-A** remained unchanged. Similarly, when a powder sample of **X-sql-1-Cu-(MeOH)$_2$•2MeOH** was exposed to air flow the transformation to **X-sql-1-Cu-A** started via applying additional heat, the conversion being completed by 373 K (Figure S12). Unlike the conversion in air and nitrogen flow, the exposure of **X-sql-1-Cu-(MeOH)$_2$•2MeOH** to dynamic vacuum resulted in rapid formation of **X-sql-1-Cu-B** at 298 K. This phase remained unchanged with further heating up to 473 K (Figure S12). In addition, exposure of **X-sql-1-Cu-A•MeOH** or **X-sql-1-Cu-B** to nitrogen flow and further heating showed no structural transformation in either sample (Figure S12) indicating that the two

polymorphs are stable to heat, air, and nitrogen once they are formed. **X-sql-1-Cu-A** was also found to be stable upon cooling to 195 K (Figure S13).

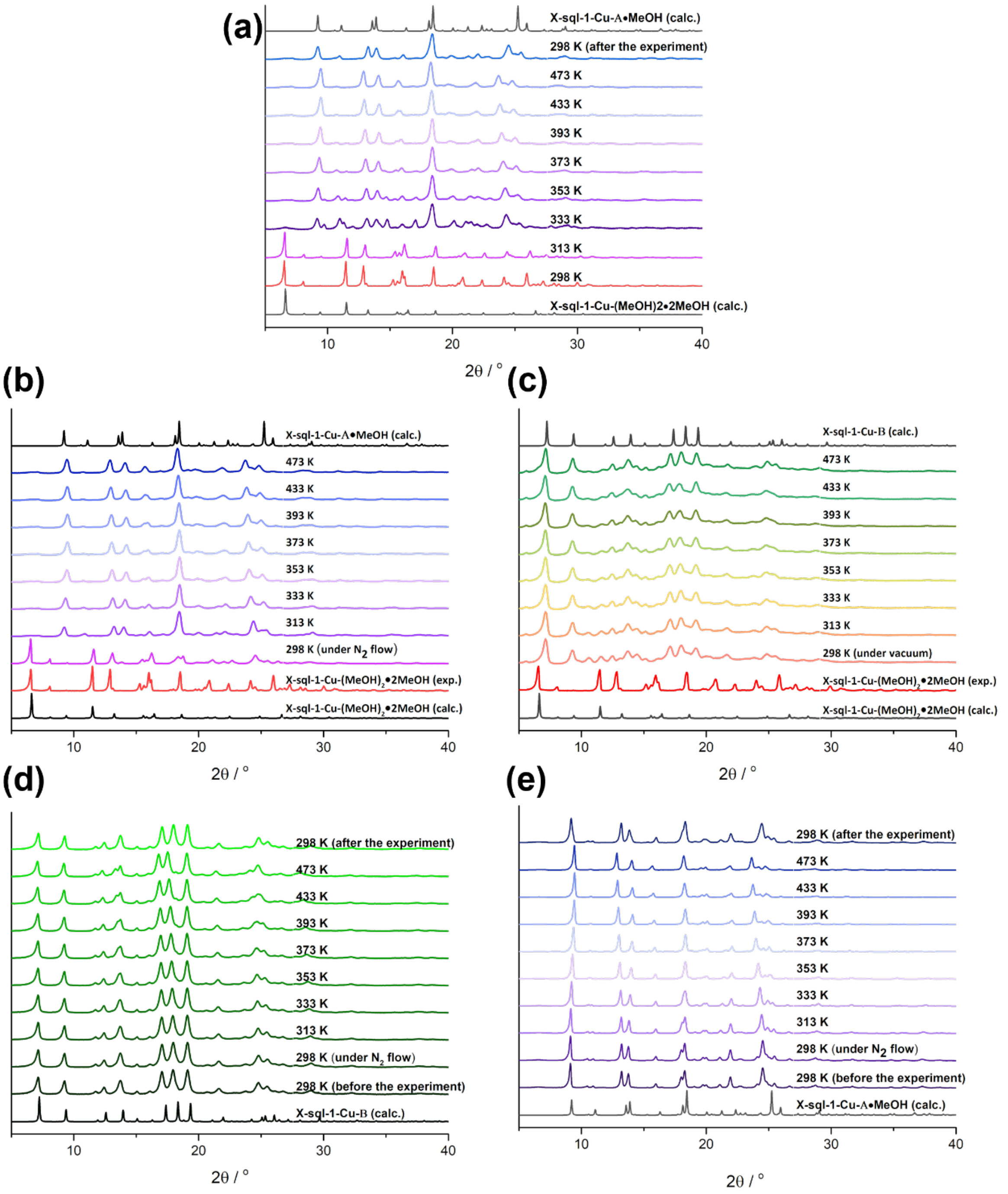

Figure. S 14. Overlay of VTPXRD for **X-sql-1-Cu** starting from a) **X-sql-1-Cu-(MeOH)$_2$•2MeOH** air flow; b) **X-sql-1-Cu-(MeOH)$_2$•2MeOH** under continuous N_2 flow c) **X-sql-1-Cu-(MeOH)$_2$•2MeOH** under

continuous dynamic vacuum; d) **X-sql-1-Cu-B** under continuous N_2 flow and e) **X-sql-1-Cu-A•MeOH** under continuous flow of N_2.

Synchrotron powder X-ray diffraction data was collected at beamline I11 at the Diamond Light Source (λ = 0.825978 Å and zero error = 0.0045). Powder sample of **X-sql-1-Cu-A•MeOH** phase was loaded in a Φ=0.5 mm quartz capillary and connected to dynamic vacuum. Sample was heated from 298 K to 333 K with further cooling to 195 K (ramp 5 K/min) using Cryostream Plus while two seconds scans every minute were collected using positional scanning detector (PSD).[59]

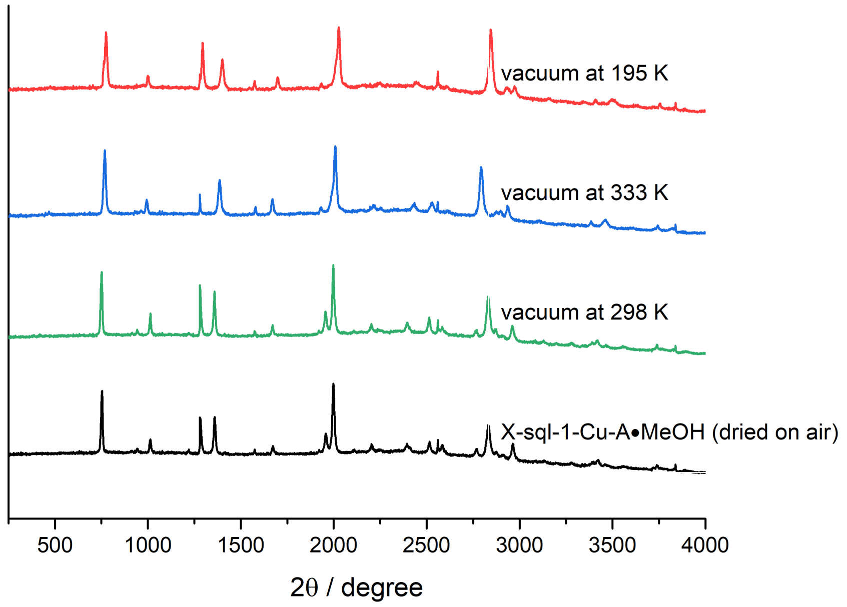

Figure S 15. VTPXRD of **X-sql-1-Cu-A•MeOH** polymorph starting from 298 K to 195 K under dynamic vacuum.

4. Polymorphs interconvertibility

1. MeOH sorption studies

Dynamic MeOH vapor sorption measurements were conducted using a Surface Measurement Systems DVS Vacuum at 298 K. Activated samples of **X-sql-1-Cu-A** were further degassed under high vacuum (1x10-4 Torr) *in situ* and stepwise increase in relative pressure were controlled by equilibrated weight changes of the sample (dM/dT = 0.01%/min) from 0 to 95%. Vacuum pressure transducers were used with the ability to measure from 1x10-6 to 760 Torr with a resolution of 0.01%. Approximately 15 mg of sample was used for MeOH sorption experiment.

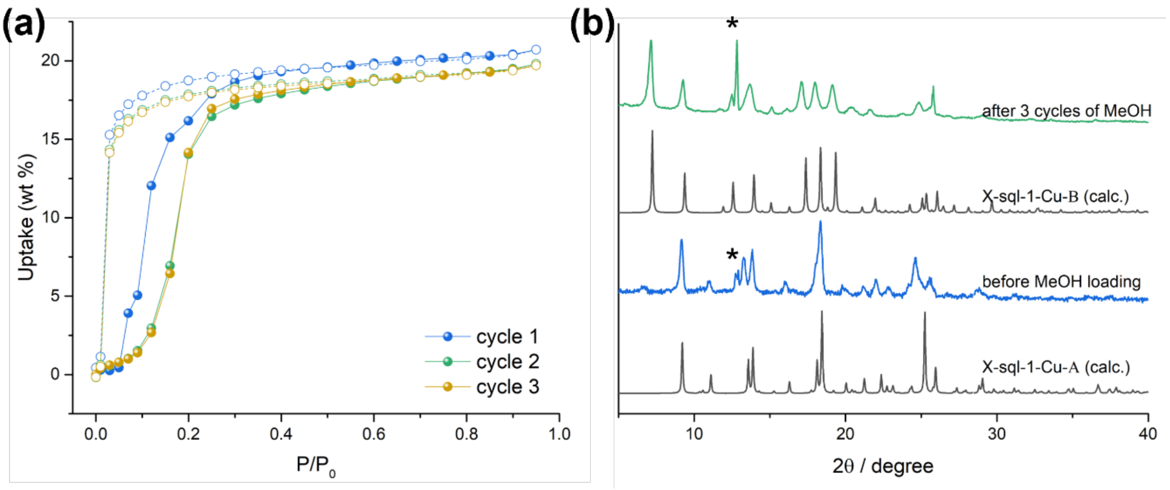

Figure S 16. a) MeOH sorption isotherms for **X-sql-1-Cu-A** and b) PXRD after 3 cycles of adsorption. Asterisk corresponds to impurity peak of 12.7 ° which came from starting materials contaminated with unidentified impurity.

2. Scanning Electron microscopy (SEM)

Scanning electron microscopy measurements were carried out on a Hitachi SU-70 instrument, using 3 kV acceleration voltage. Before the measurement, the samples were dispersed on carbon tape attached to SEM stubs and were gold-coated for 50 seconds to enhance surface conductivity. To determine the crystal length and width 104 particles for both non-ground and ground of **X-sql-1-Cu-A** phase were analysed by using ImageJ software.

(a) (b)

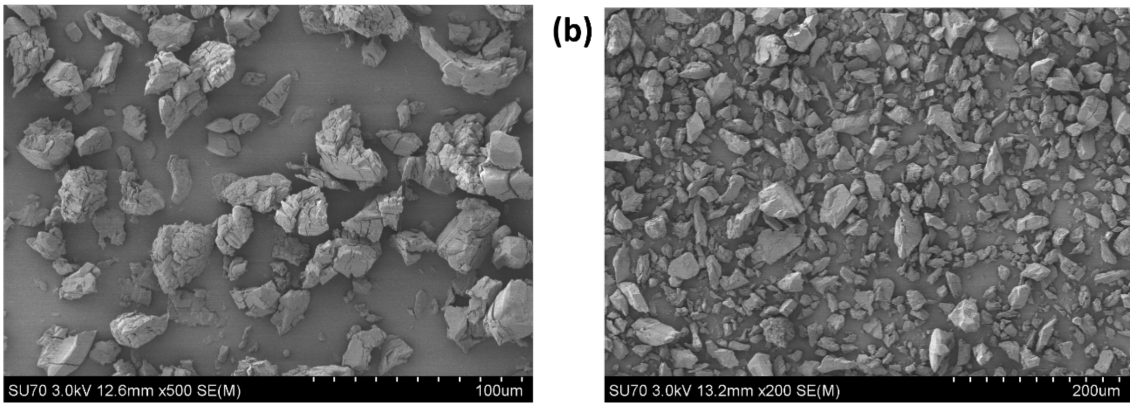

Figure S 17. SEM images of **X-sql-1-Cu-A (**a) non-ground and (b) ground.

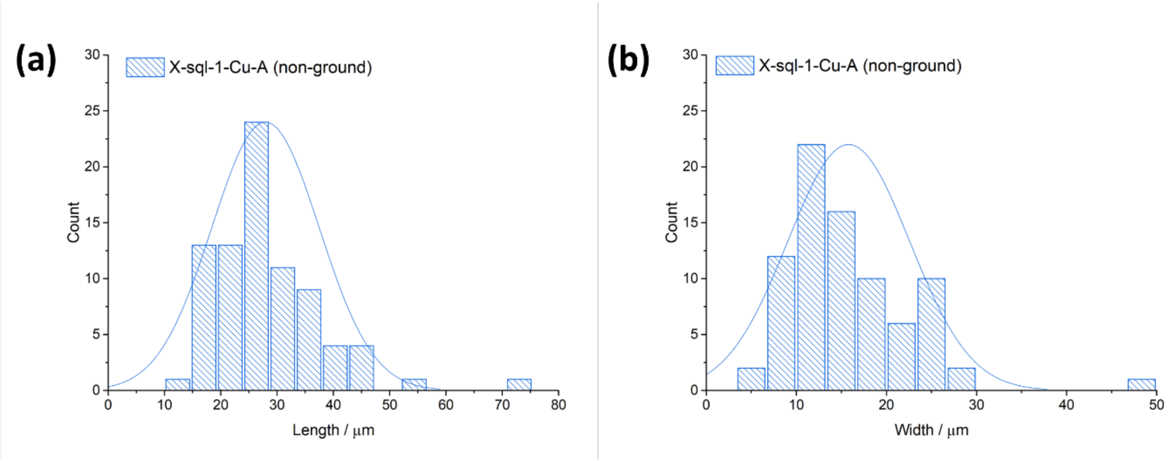

Figure S 18. Crystal size distributions for non-ground sample of **X-sql-1-Cu-A**: (a) crystal length and (b) crystal width.

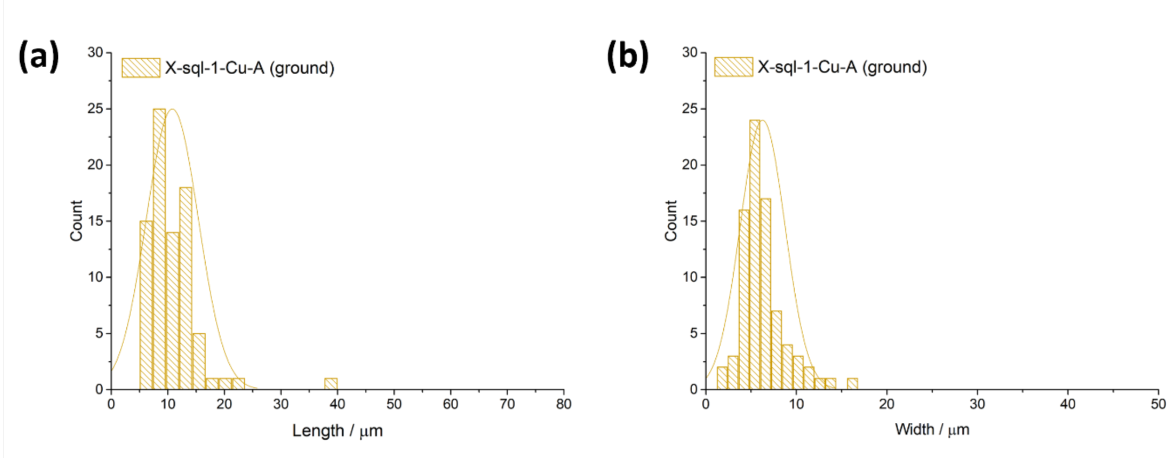

Figure S 19. Crystal size distributions for ground sample of **X-sql-1-Cu-A**: (a) crystal length and (b) crystal width.

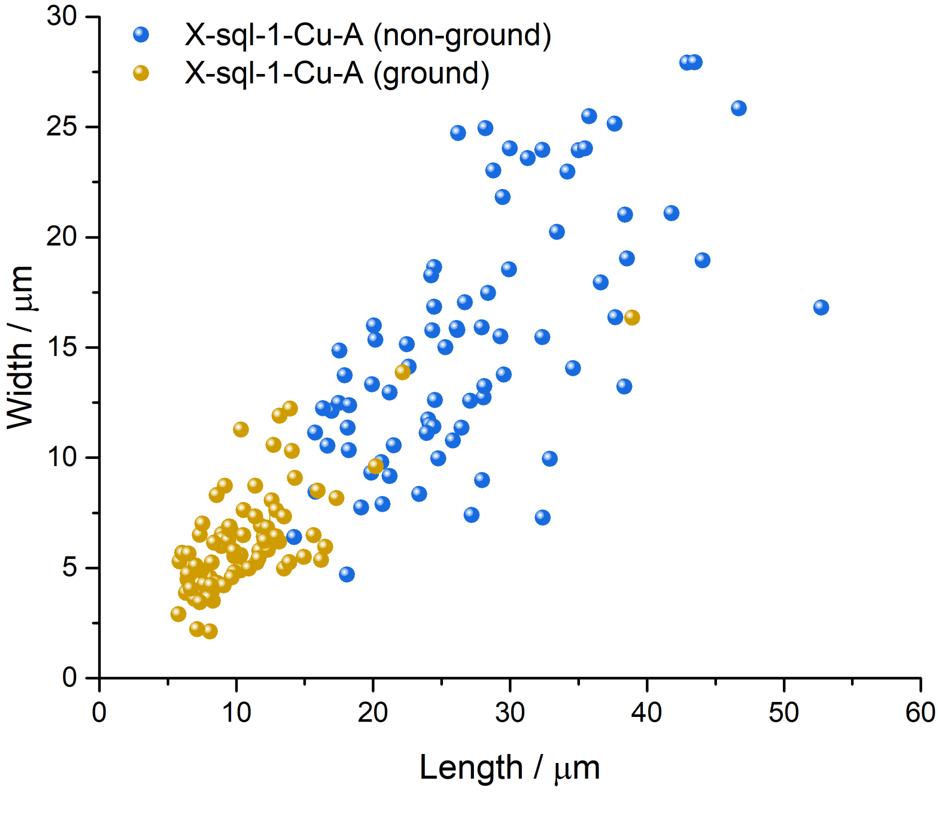

Figure S 20. Crystal width plotted against crystal length for non-ground (blue) and ground (orange) of **X-sql-1-Cu-A**.

Table S 5. *Particle size distribution of crystal length and crystal width determined from SEM images of non-ground and ground sample of* ***X-sql-1-Cu-A****.*

			σ (μm)
**X-sql-1-Cu-A (non-ground)**	Length	27.9	9.6
	Width	15.8	6.8
**X-sql-1-Cu-A (ground)**	Length	10.8	4.7
	Width	6.3	2.5

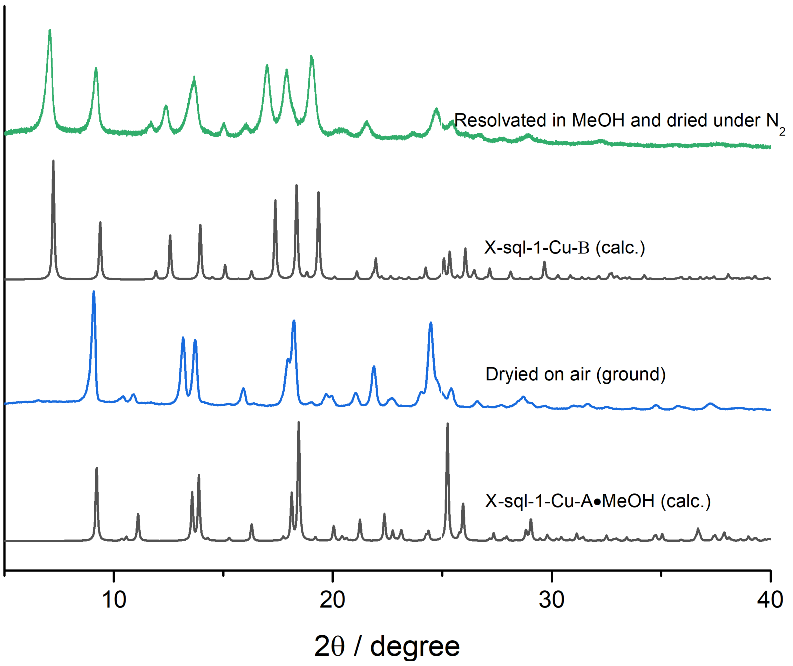

Figure S 21. PXRD of grounded **X-sql-1-Cu-A** and its MeOH resolvation.

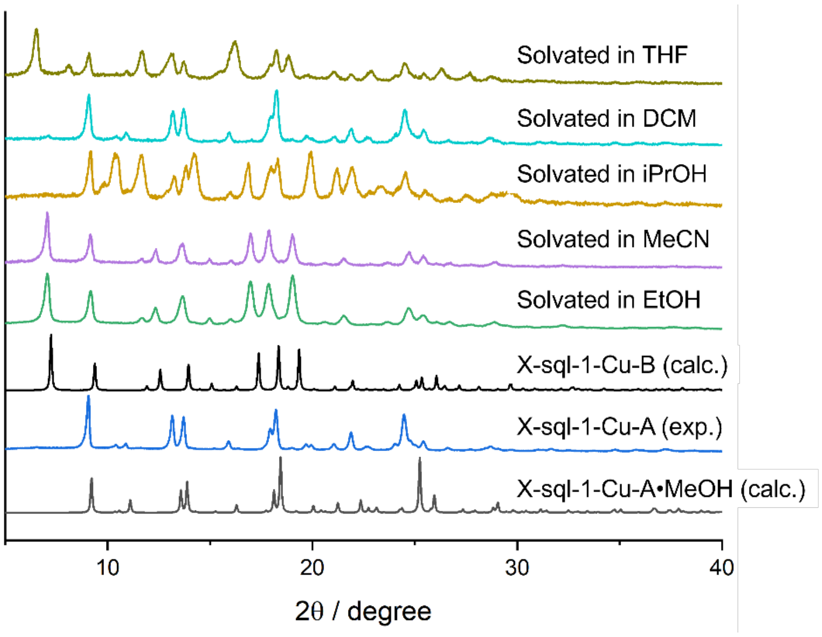

Figure S 22. PXRD patterns of **X-sql-1-Cu-A** solvated in different solvents and dried under N_2 flow.

3. Water vapor sorption

Water vapor sorption isotherm determination was performed using Adventure Dynamic Vapour Sorption (DVS) instrument manufactured by Surface Measurement Systems. The instrument gravimetrically measures vapor uptake using air as a carrier gas. Digital mass flow controllers regulate flows of dry and saturated gases. Relative humidity is generated by precisely mixing dry and saturated gas flows in desired flow ratios which produce expected relative humidity. Temperature was maintained at 300 K by enclosing the system in a temperature-controlled incubator. The mass of the sample was determined by a high-resolution microbalance Ultrabalance Low Mass with a precision of 0.01 µg. Microbalance has symmetric configuration with two branches of the balance being exposed to the same gas and being kept at the same temperature, which allows negation of buoyancy and drag effects. 400 sccm (Standard Cubic Centimeters per Minute) flow was used for the measurements at 300 K. Prior to the measurement, the sample was in-situ activated in dry air at 333 K for 60 minutes using built-in preheater and consequently cooled to sorption temperature in 90 minutes. For each isotherm point, $dm/dt < 0.01$ % min^{-1} was used as criteria of reaching equilibrium.

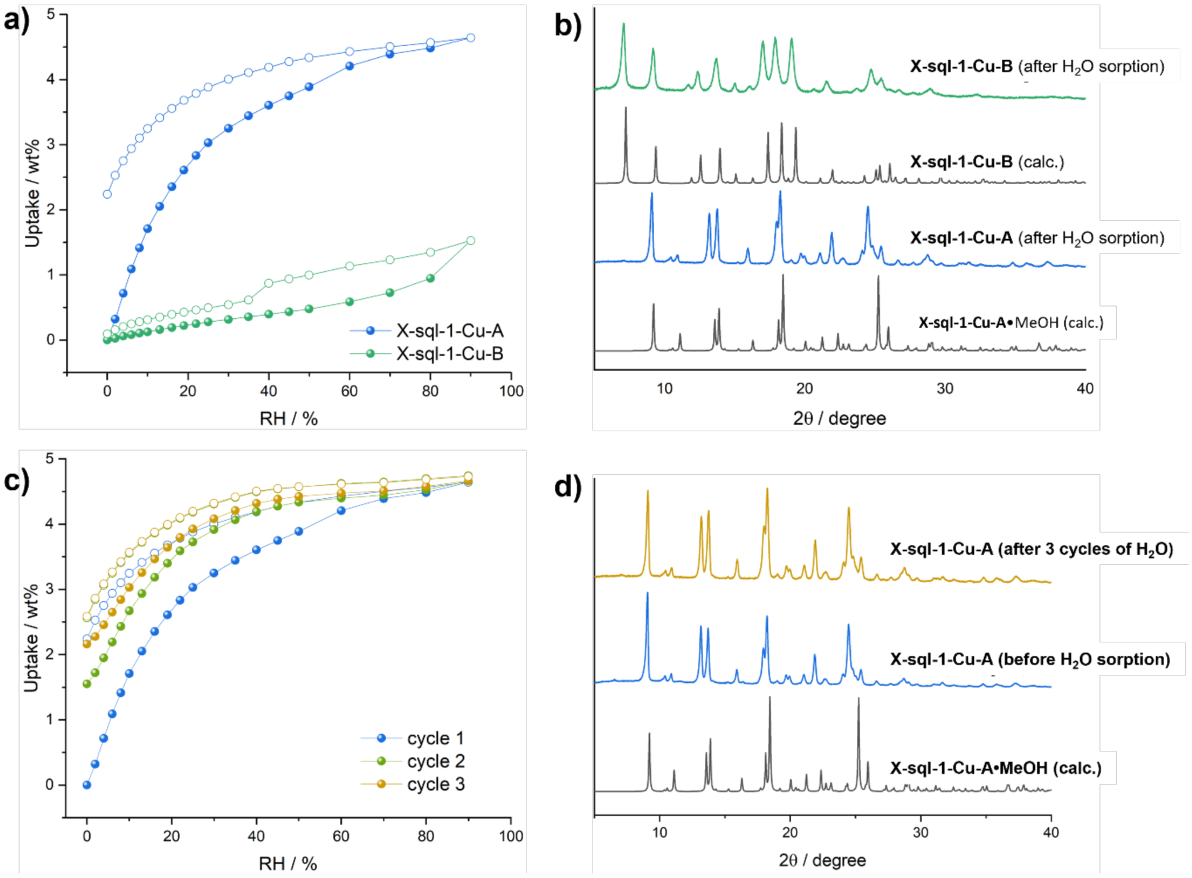

Figure. S 23. a) Water vapor sorption isotherms for **X-sql-1-Cu** at 300 K; b) corresponding PXRD patterns for both **X-sql-1-Cu-A** and **X-sql-1-Cu-B** for the water sorption experiment; c) cycling water vapor sorption for **X-sql-1-Cu-A** collected at 300 K and d) PXRD patterns for **X-sql-1-Cu-A** before and after cycling water sorption experiment.

4. Low Pressure Gas sorption

Prior to sorption experiments, a sample of **X-sql-1-A** was obtained by activation of freshly prepared **X-sql-1-A•MeOH** on a SmartVacPrep™ using dynamic vacuum and heating for 10 h at 333 K. High grade gases were used as obtained from BOC Gases Ireland Ltd.: CO_2 (99.999 %), N_2 (99.9995 %), C_2H_2 (98.5 %).

A Micromeritics 3Flex surface area and pore size analyzer 3500 was used for collecting the 273 K and 298 K sorption isotherms of CO_2 and C_2H_2 as well as 195 K isotherms of CO_2 and 77 K N_2 isotherms on **X-sql-1-A**. The temperature at 195 K and 77 K were maintained using a 4 L Dewar filled with a dry ice-acetone mixture and a liquid nitrogen bath respectively. Bath temperatures of 273 and 298 K were precisely controlled with a Julabo ME (v.2) recirculating control system containing a mixture of ethylene glycol and water.

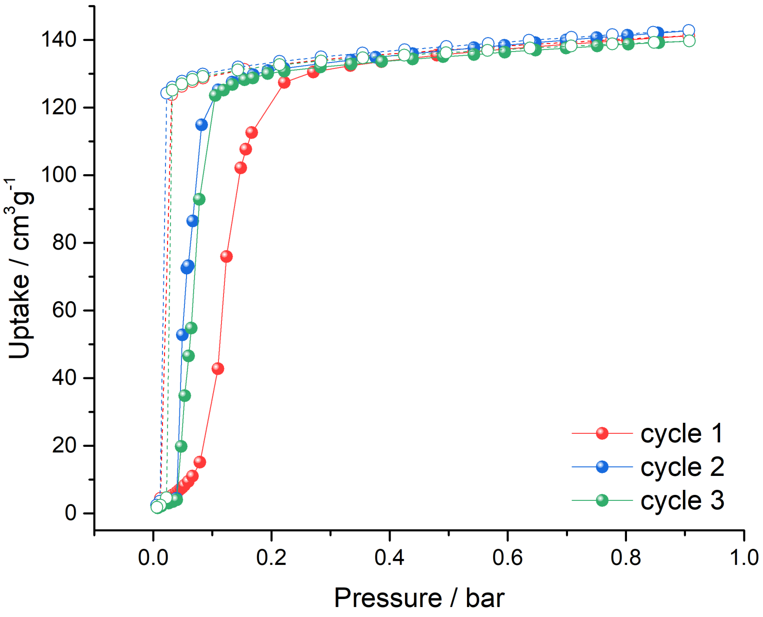

Figure S 24. Three cycles of CO_2 sorption/desorption starting with **X-sql-1-Cu-A** at 195 K.

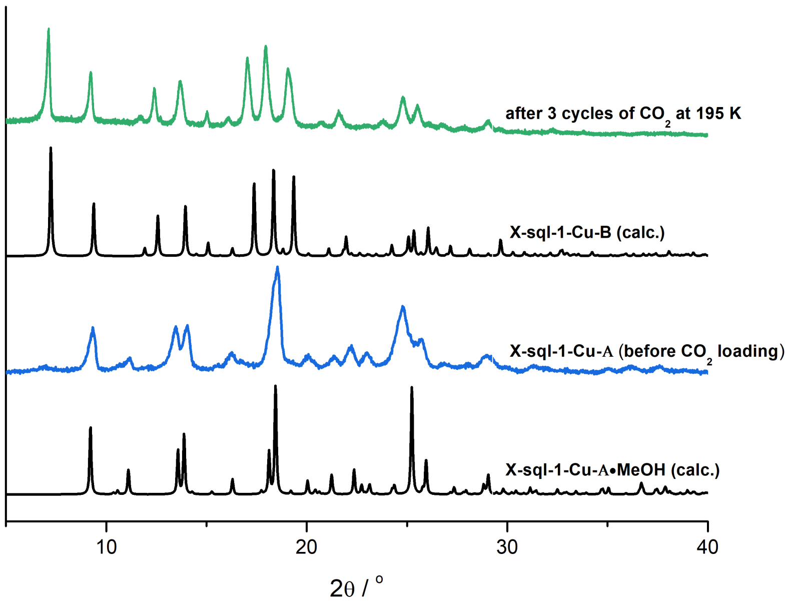

Figure. S 25. PXRD after 1 bar CO_2 sorption of **X-sql-1-Cu-A** at 195 K

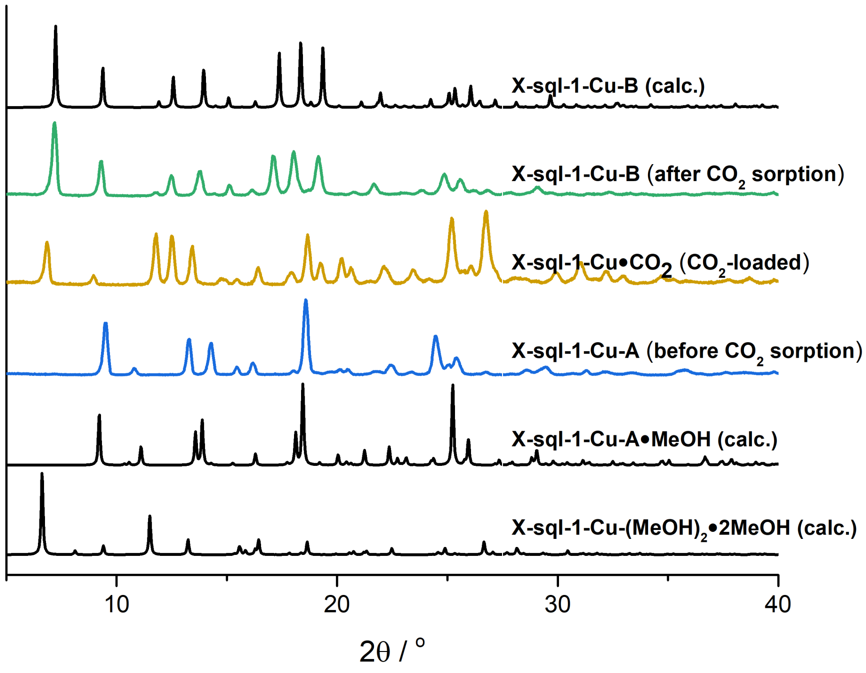

Figure S 26. PXRD patterns of CO_2-loaded phase, **X-sql-1-Cu•CO₂.**

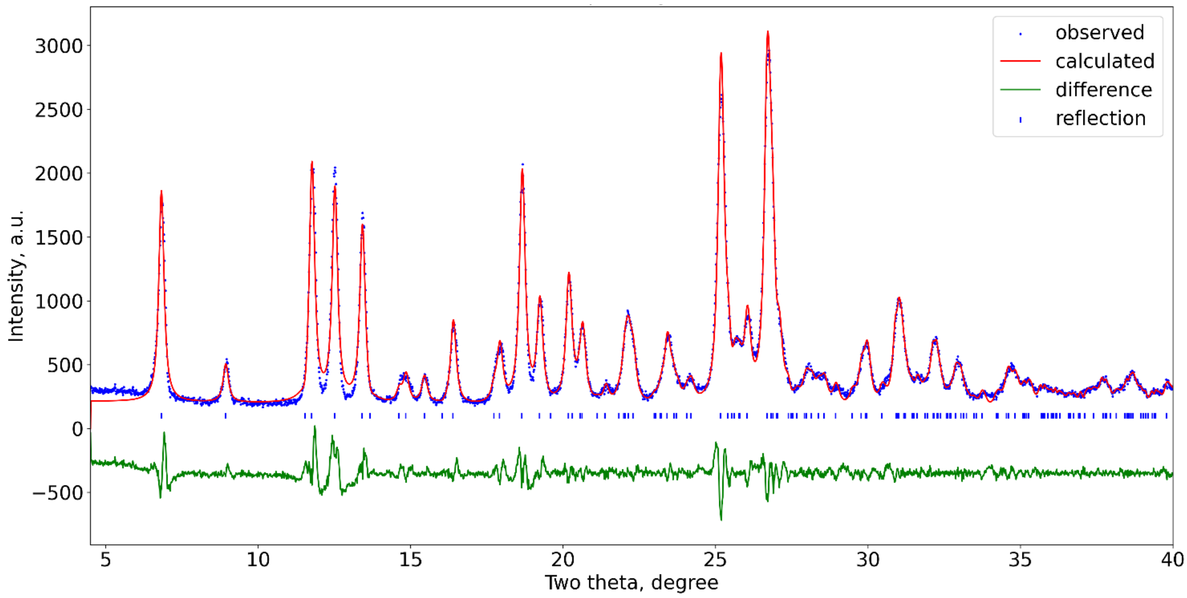

Figure S 27. Pawley profile fit of **X-sql-1-Cu•CO_2** at 195 K. Unit cell parameters are: SG=P 21/c, a=13.227(6)Å, b=15.333(15)Å, c=8.817(3)Å, beta=101.74(15)°, volume=1750.6(3)$Å^3$, Rwp = 1.13%.

The unit cell parameters of CO_2 loaded **X-sql-1-Cu•CO_2** were determined from the powder X-ray diffraction patterns collected at 195 K. Positions of the first 18 peaks were used for indexing using EXPO2014.[60] Pawley profile fit of powder X-ray diffraction pattern was performed using GSASII.[61]

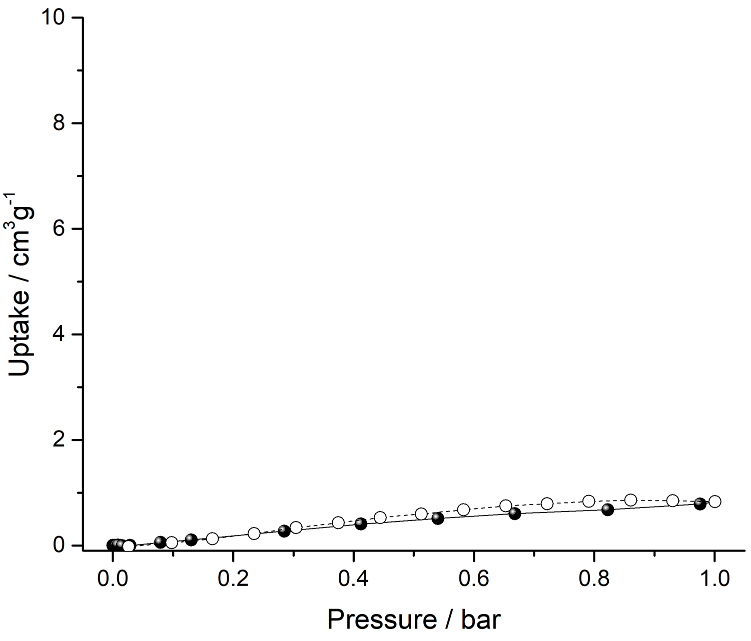

Figure S 28. CO_2 298 K sorption experiment of **X-sql-1-Cu-B**.

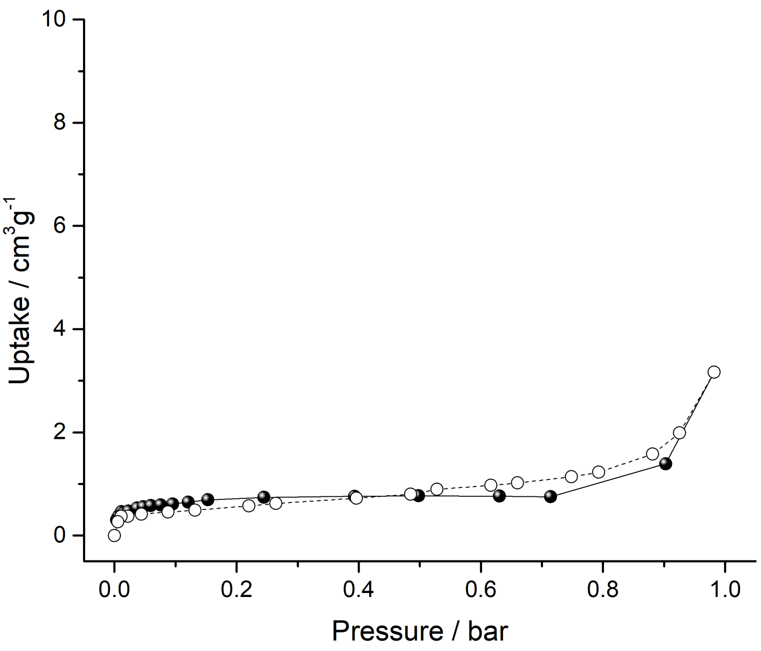

Figure S 29. N_2 sorption at 77 K of **X-sql-1-Cu-B**.

5. *In situ* Differential Scanning Calorimetry (DSC)

In situ Differential Scanning Calorimetry (DSC) analyses were carried out on TA Instrument DSC Q20 from 198 K to 298 K at a scan rate of 5 K min^{-1}. 10 mg of the **X-sql-1-Cu-A** was activated at 333 K for 2 hours under the continuous flow of N_2 followed by CO_2 dosing.

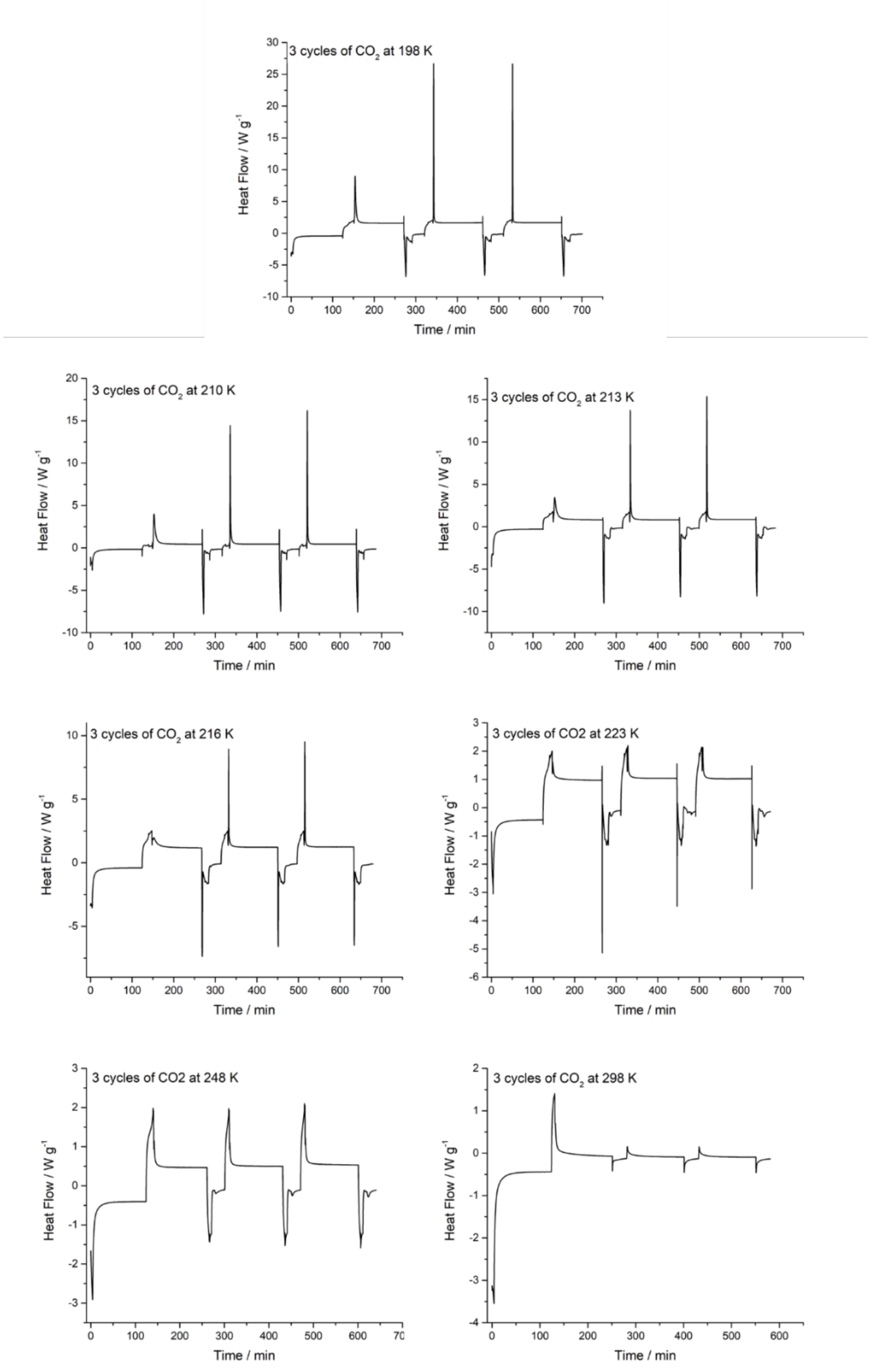

Figure S 30. In situ DSC experiment of exposing **X-sql-1-Cu-A** to CO_2 at different temperature.

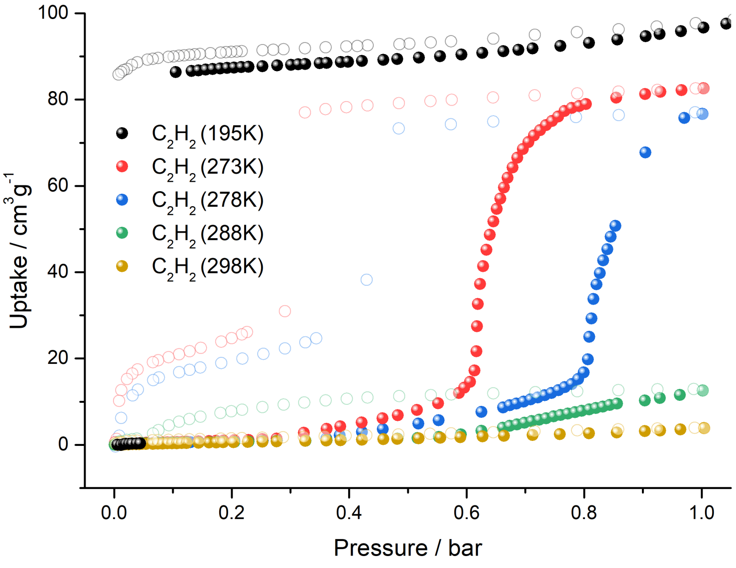

Figure S 31. C_2H_2 gas sorption isotherms collected at different temperatures for **X-sql-1-Cu-B**.

6. *In situ* Powder X-ray Diffraction

In situ PXRD experiments in parallel to gas adsorption were performed on a specialized Empyrean powder X-ray diffractometer (ω–2θ goniometer, K-Alpha1 system) using a customized setup based on an ARS DE-102 closed cycle helium cryostat, which assured isothermal conditions in the temperature range of 195 ± 0.1 K. The adsorption cell is based on a 1.33″ CF-flange and is covered with a beryllium dome. The cell was connected to the low-pressure port of the BELSORP-max volumetric adsorption instrument. The TTL trigger was used for establishing the communication between BELSORP-max and Empyrean software and ensure the measurement of the adsorption isotherm and PXRD pattern data collection in a fully automated mode. The diffraction experiments were performed using ω–2θ scans in transmission geometry in the range of 2θ = 2-70°. The parallel linear Cu Kα1 beam, generated by the hybrid 2xGe(220) monochromator, 4 mm mask, and primary divergence and secondary antiscatter slits with 1/4° opening were used for data collection. A Pixcel-3D detector in 1D scanning mode (255 active channels) was used. A complete 3 cycles of CO_2 adsorption–desorption isotherms at 195 K for

X-sql-1-Cu-A were measured on 13 mg of the sample and PXRD patterns were in situ recorded after equilibration (0.1% of pressure change within 300 s) at selected points of the isotherm.

5. Computational methodology

1. Density Functional Theory calculations

Periodic Density Functional Theory (DFT) calculations were performed using the projected augmented wave (PAW) formalism[62] as implemented in the Vienna Ab Initio Simulation Package (VASP 5.4.4),[63, 64] employing the BEEF-vdW exchange-correlation functional.[65] The atomic positions in the structures of **X-sql-1-Cu** were optimized at their experimentally refined cell parameters, *i.e.* the closed pore form **X-sql-1-Cu-A** (a = 19.358 Å, b = 17.059 Å, c = 18.165, α = γ = 90°, β = 97°) and the closed pore form **X-sql-1-Cu-B** (a = 24.478 Å, b = 14.857 Å, c = 11.014, α = γ = 90°, β = 95.775^o). Furthermore, we also constructed an open-pore form of these closed pore forms based on the cell parameters of the fully CO_2-loaded phase (a = 13.227, b = 15.3331, c = 8.81743, α = γ = 90°, β = 101.7^o), to generate the open pore forms **X-sql-1-Cu-A•CO_2** and **X-sql-1-Cu-B•CO_2** with the same crystal symmetry as their corresponding closed pore forms. These four structures (**-A, -B, -A•CO_2** and **-B•CO_2**, see **Table S6**) were optimized at the Γ-point using the conjugate gradient algorithm with force and electronic convergence criteria of 0.01 eV/Å and 10^{-5} eV, respectively, a Gaussian smearing of 0.05 eV, and an energy cutoff of 500 eV. Subsequently, two Nudged Elastic Band (NEB) runs were performed after generating 8 images between the closed pore forms (**X-sql-1-Cu-A** and **X-sql-1-Cu-B**) and their constructed open-pore form analogues (**X-sql-1-Cu-A•CO_2** and **X-sql-1-Cu-B•CO_2**) maintaining the symmetry of the original closed pore form during the NEB simulation, with force convergence criterion set to 0.03 eV/Å and spring constant of 5 eV/Å^2 between subsequent images. The unit cell parameters for the intermediate images in these NEB runs were found via linear interpolation and were kept fixed. When the NEB was converged, all structures were converged tighter, employing the conjugate gradient algorithm with force and electronic convergence criteria of 0.01 eV/Å and 10^{-6} eV, respectively, and a 3x3x3 Monkhorst-Pack [66, 67] k-point grid. The structures were ranked according to possible initial CO_2 adsorption from **X-sql-1-Cu-A** and subsequent desorption pathways, *i.e.* from **-A**, to **-A•CO_2,** and **-B**, to **-B•CO_2.** The potential phase transition from an **-A•CO_2** to **-B•CO_2** intermediate structure through

CO_2 desorption is energetically favorable, since the **X-sql-1-Cu-B** based intermediate empty host structures are lower in energy than the **X-sql-1-Cu-A** based intermediate structures (Figure 5). To investigate this transition, grand canonical Monte Carlo (GCMC) simulations were performed on all structures., and the maximum uptake at a CO_2 partial pressure of 1 bar presented in Table S6.

Table S6. *Cell parameters of structures employed in DFT calculations and the generated structures for further CO2 sorption studies (labeled 0 (**-A**), 1, 2, …, 9 (**-A•CO₂**), 10(**-B•CO₂**), …, 18, **-B**).*

**Structure number**	**a (Å)**	**b (Å)**	**c (Å)**	**α (°)**	**β (°)**	**γ (°)**	**Volume (Å³)**		**Max. uptake at pCO₂ = 1 bar (GCMC)**
**-A**	19.358	17.059	18.165	90	97	90	1486.13	**-A**	0
**1**	20.1464	16.8672	18.10176	90	98.1371	90	1522.33		0.03
**2**	20.9348	16.6755	18.03956	90	98.5773	90	1556.79		68.42
**3**	21.7234	16.4837	17.97844	90	99.0204	90	1589.53		68.42
**4**	22.5118	16.292	17.91842	90	99.4666	90	1620.57		111.18
**5**	23.3002	16.1002	17.85948	90	99.9158	90	1649.92		136.84
**6**	24.0886	15.9084	17.80164	90	100.368	90	1677.60		136.84
**7**	24.8772	15.7167	17.74492	90	100.823	90	1703.66		136.84
**8**	25.6656	15.5249	17.68932	90	101.281	90	1728.06		136.84
**9**	26.454	15.3331	17.63486	90	101.742	90	1750.86	**-A•CO₂**	136.84
**10**	26.454	15.3331	17.63486	90	101.742	90	1750.86	**-B•CO₂**	66.16
**11**	26.2344	15.2802	17.44996	90	101.133	90	1715.86858		5.573
**12**	26.0148	15.2273	17.26706	90	100.511	90	1681.32807		0
**13**	25.7954	15.1744	17.08624	90	99.8765	90	1647.23585		0
**14**	25.5758	15.1215	16.90758	90	99.2282	90	1613.57068		0
**15**	25.3562	15.0686	16.7311	90	98.5661	90	1580.33682		0
**16**	25.1366	15.0157	16.55692	90	97.89	90	1547.5362		0
**17**	24.9172	14.9628	16.3851	90	97.1996	90	1515.17739		0
**18**	24.6976	14.9099	16.2157	90	96.4947	90	1483.23187		0
**-B**	24.478	14.857	16.0488	90	95.775	90	1451.70992	**-B**	0

2. Grand Canonical Monte Carlo simulations

Grand Canonical Monte Carlo (GCMC) simulations to gain deeper insight into CO_2 adsorption and the **X-sql-1-Cu-A•CO₂** to **X-sql-1-Cu-B•CO₂** phase transformation at 195 K within a (2x1x2) supercell of Cu(Imibz)₂. GCMC simulations were conducted in Material Studio[68] utilizing the COMPASS force field[69, 70]. The frameworks and CO_2 point charges were assigned using the COMPASS force field. The point charges [e] for CO_2 molecules are provided in Figure S34.

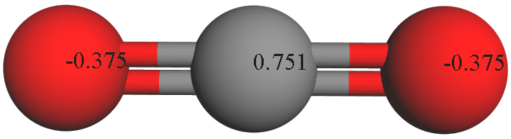

Figure S32. Water molecule model with atomic point charges according to COMPASS II force field.

In the grand canonical ensemble, the Metropolis sampling method considered various moves: translation (which involves translating the center-of-mass of the selected adsorbate molecule), rotation (rotating the selected adsorbate molecule), regrowth (removing a selected adsorbate molecule from the system and reintroducing it at a random position with random orientation), and conformer (collecting multiple sorbate conformations). These moves were assigned relative probabilities of 1, 1, 0.1, and 1, respectively. Additionally, a cutoff distance of 7.4 Å was applied. Each GCMC simulation comprised 1×10^6 equilibration steps, succeeded by 1×10^6 production steps to ensure reasonable ensemble averages. During the GCMC simulations, the unit cells and framework atoms were maintained fixed at their DFT-optimized positions. The adsorption isotherms were simulated, and CO_2 uptake plotted versus pressure for all considered structures in Table S6. Figure S33 shows the simulated GCMC adsorption isotherms for all studied **X-sql-1-Cu-A** and **X-sql-1-Cu-B** -based structures (see Table S6). Notwithstanding the fact that the experimentally observed CO_2 adsorption isotherms cannot be aligned with a single simulated isotherm due to the rigidity of each host structure, tracking the matching points between the experimental and all simulated isotherms enables the prediction of the most plausible unit cell volume and phase transformation pathways during the adsorption and subsequent desorption process. Figure S33 (a) shows empty framework representations during CO_2 adsorption from the **X-sql-1-Cu-A** structure and experimental adsorption isotherm, while the **X-sql-1-Cu-B** based simulated isotherms are displayed with the experimental desorption isotherm in Figure S33 (b). The gap in uptake between the most open structures 9 (**-A•CO_2**) and 10 (**-B•CO_2**) based on the experimental CO_2-loaded structure (Figure S33 (b)) shows that full uptake can only be reached in the **-A•CO_2**-based structure 9. Subsequently, the CO_2 desorption from 9 (**-A•CO_2**) will easily transform the loaded **X-sql-1-Cu-A**-based intermediates (e.g., 5-9) into lower energy **X-sql-1-Cu-B** based intermediate structures (e.g., 10-14, Figure S33) for unit cell volumes over 1650 $Å^3$. The most plausible frameworks in the adsorption-desorption process are represented in Figure S34.

Using the simulated isotherms, a contour plot and 3-dimensional CO_2 uptake representing each simulated structure (unit cell volume) with respect to pressure was constructed (Figures S35-S37). Interestingly, the most plausible experimental adsorption-desorption trajectory can be visualized on these plots by correlating the experimental uptake with the simulated isotherms to determine the most plausible unit cell volume at various experimental isotherms points.

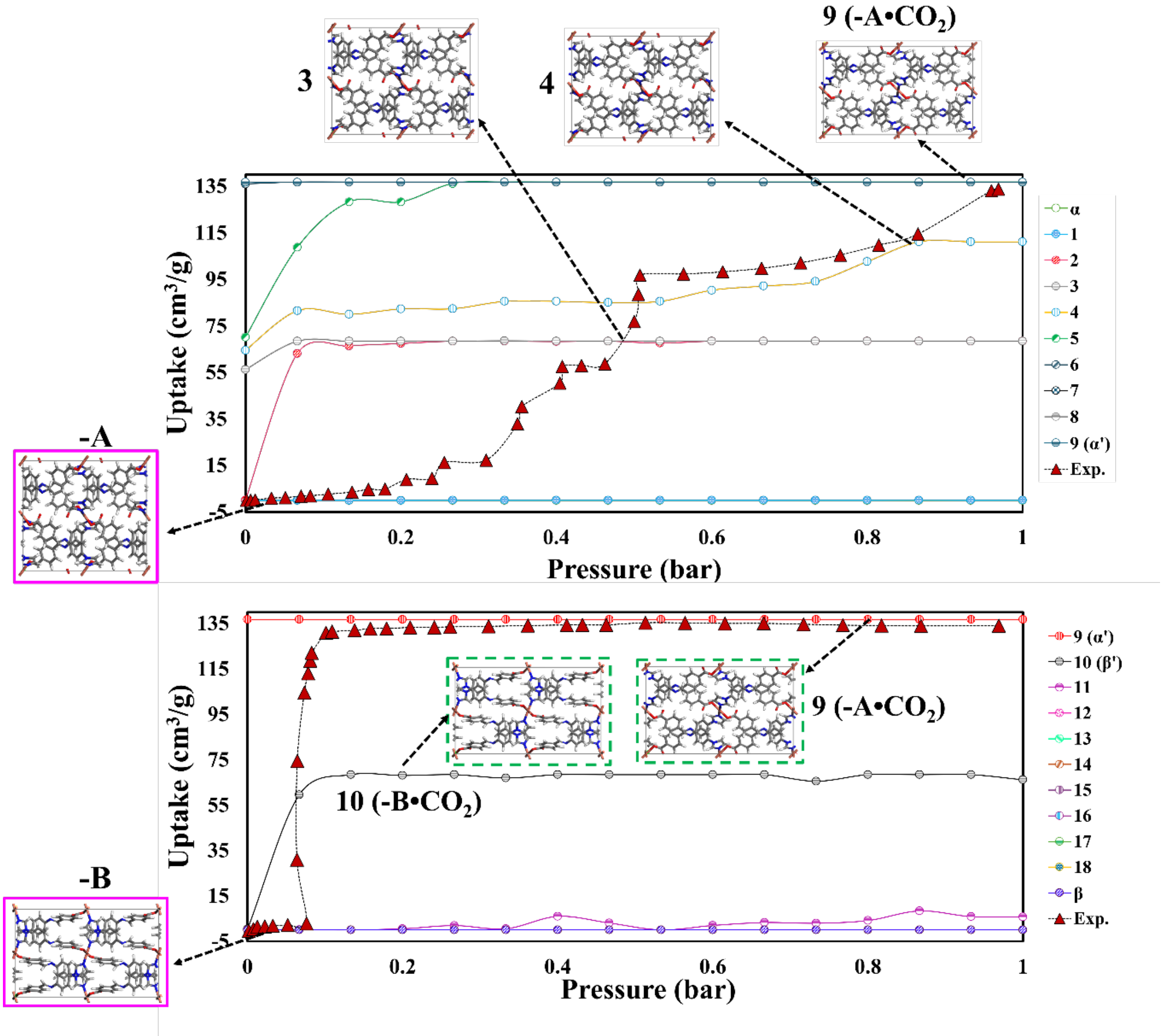

Figure S 33. GCMC simulated CO_2 adsorption isotherms for (a) **X-sql-1-Cu-A**-based structures (**-A**, 1,2,…, and 9(**-A•CO$_2$-9**)), and (b) **-B**-based structures (10 (-**B•CO$_2$-10**)), 12,…,18, and **X-sql-1-Cu-B**), and structure 9 for comparison. The labels represent the structures while the red triangulars represent the experimental data for (a) adsorption and (b) desorption.

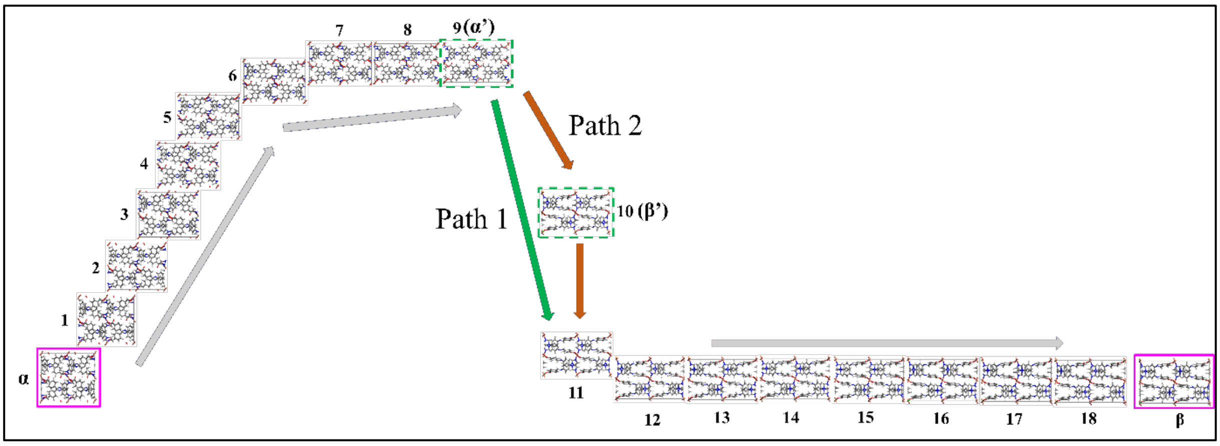

Figure S34. Potential phase transformation pathways during the adsorption-desorption process.

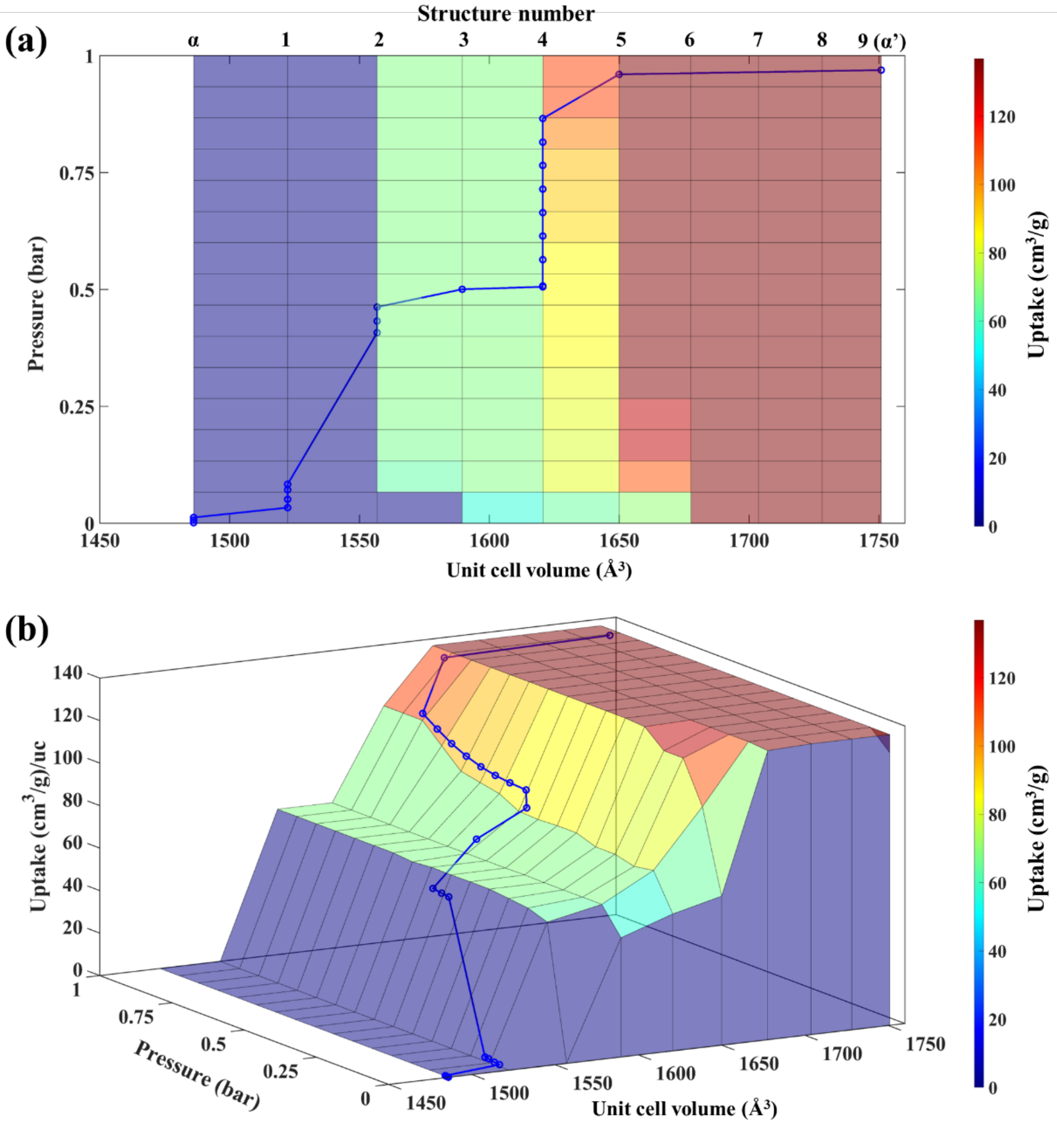

Figure S35. (a) contour plot and (b) 3-D plot representing the GCMC simulated CO_2 adsorption isotherms (uptake is color coded) for each of **X-sql-1-Cu-A**-based structures. The blue circles/lines represent the unit cell volume of most probable structure at the specified experimental uptake and CO_2 pressure.

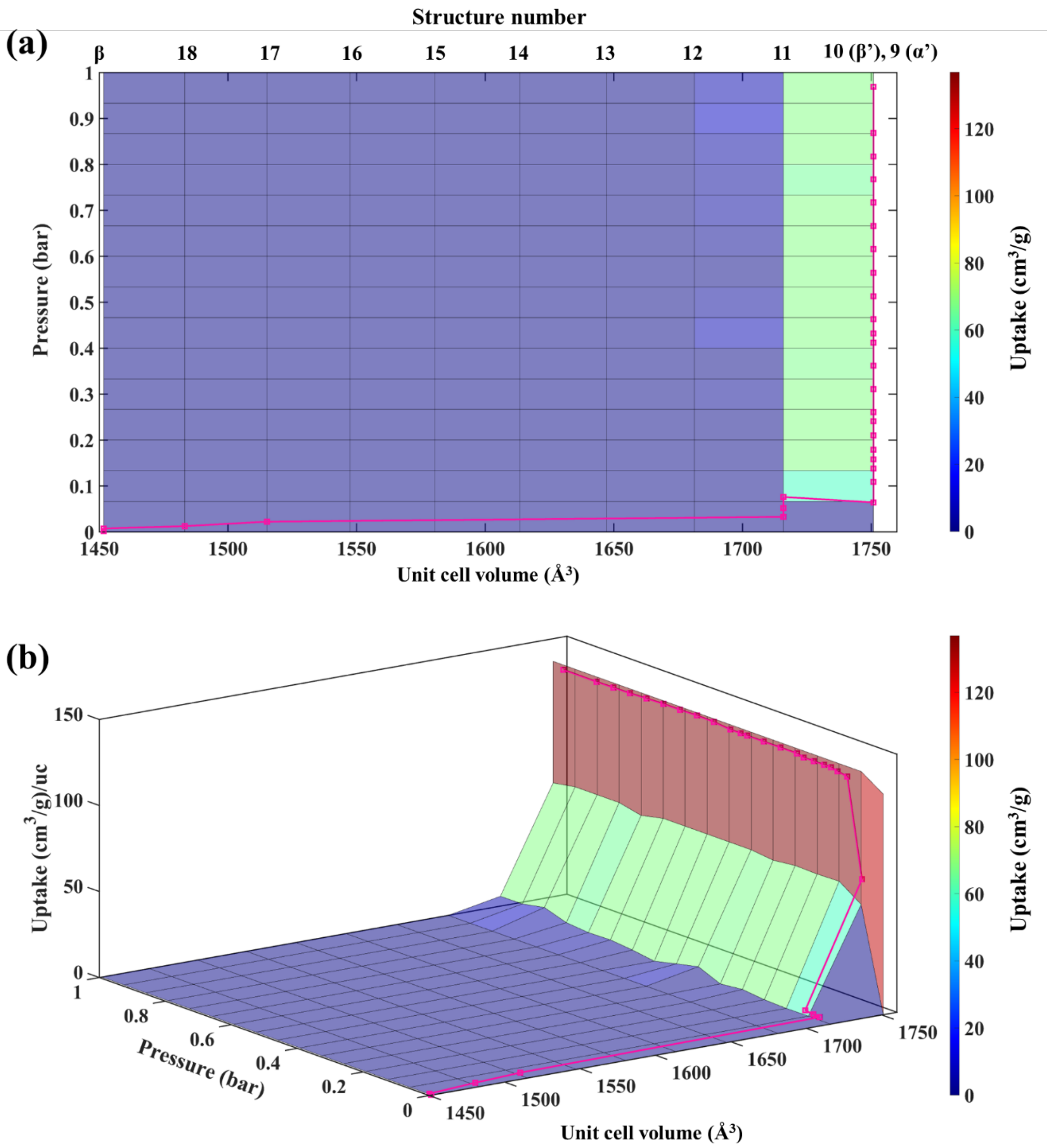

Figure S36. (a) contour plot and (b) 3-D plot representing the GCMC simulated CO_2 adsorption isotherms (uptake is color coded) for the **X-sql-1-Cu-B**-based structures and **X-sql-1-Cu-A**. The pink circles/lines represent the unit cell volume of most probable structure during desorption/adsorption at the specified experimental uptake and CO_2 pressure.

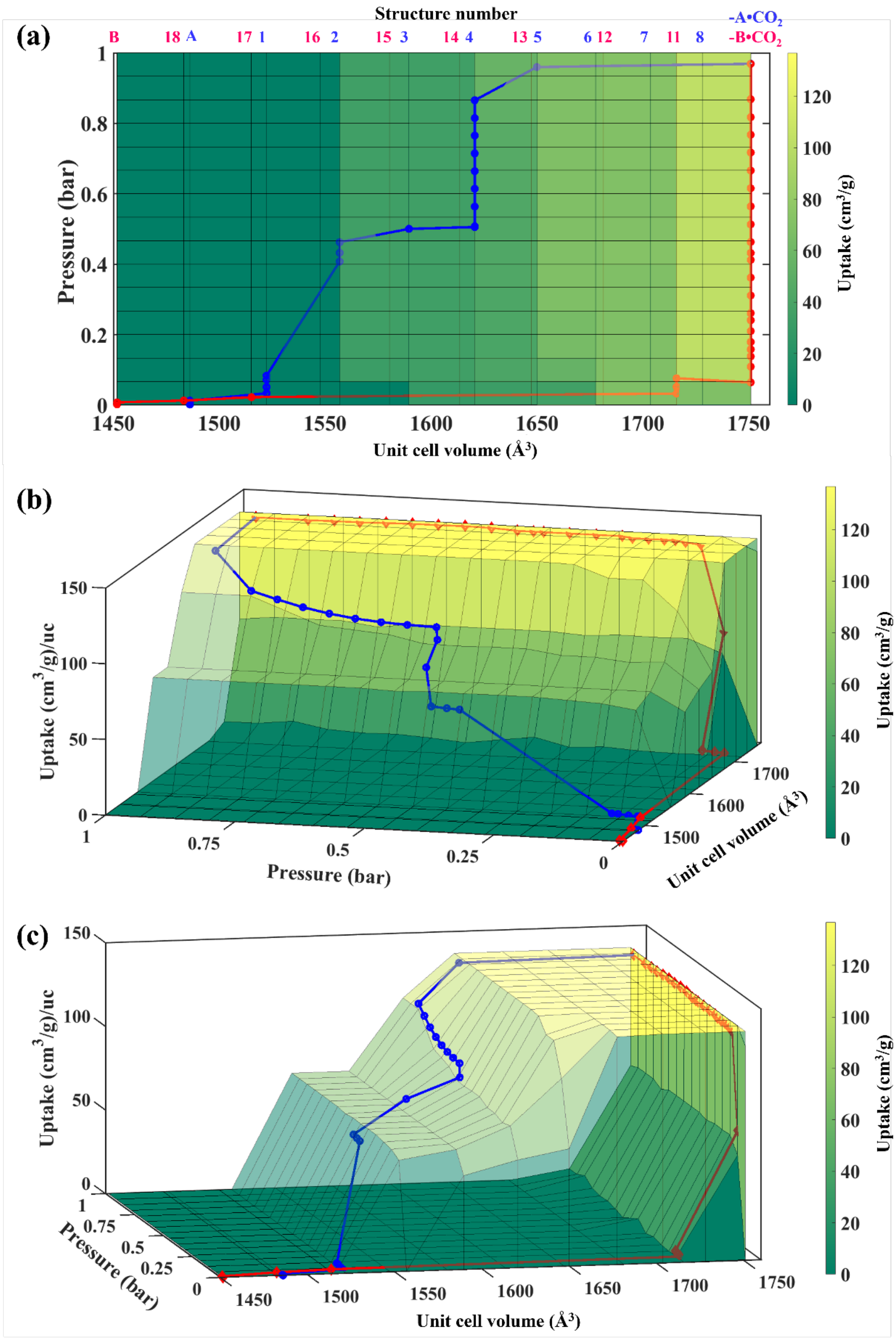

Figure S37. Contour and 3D plots representing GCMC simulated CO_2 adsorption isotherms for all the structures. The blue circles/lines and red diamonds/lines represent the unit cell volume of most

probable structure at the specified experimental uptake and pressure for **X-sql-1-Cu-A** and **X-sql-1-Cu-B**-based structures, respectively. (a), (b), and (c) panels show the graph from three views.

6. References

1. V. A. Blatov, A. P. Shevchenko and D. M. Proserpio, Applied Topological Analysis of Crystal Structures with the Program Package ToposPro, *Crystal Growth & Design*, 2014, **14**, 3576-3586.
2. C. R. Groom, I. J. Bruno, M. P. Lightfoot and S. C. Ward, The Cambridge Structural Database, *Acta Crystallographica Section B*, 2016, **72**, 171-179.
3. K. J. Nordell, K. N. Schultz, K. A. Higgins and M. D. Smith, Hydrothermal synthesis of two new lead-containing coordination polymers: 2 ∞ [PbCl2(4,4′-bipy)] and 2 ∞ [Pb2(NO3)4(H2O)2(4,4′ -bipy)2](4,4′-bipy)2, *Polyhedron*, 2004, **23**, 2161-2167.
4. C. Hu and U. Englert, Space Filling Versus Symmetry: Two Consecutive Crystal-to-Crystal Phase Transitions in a 2D Network, *Angewandte Chemie International Edition*, 2006, **45**, 3457-3459.
5. J. Darriet, W. Massa, J. Pebler and R. Stief, 4,4′-bipyMnF3, a modulated hybrid layer structure with 1D magnetic properties, *Solid State Sciences*, 2002, **4**, 1499-1508.
6. N. Masciocchi, P. Cairati, L. Carlucci, G. Mezza, G. Ciani and A. Sironi, Ab-initio X-ray powder diffraction structural characterization of co-ordination compounds: polymeric [{MX2(bipy)}] complexes (M = Ni or Cu; X = Cl or Br; bipy = 4,4′-bipyridyl), *Journal of the Chemical Society, Dalton Transactions*, 1996, 2739-2746.
7. W. Clegg, I. R. Little and B. P. Straughan, Orthorhombic anhydrous zinc(II) propionate, *Acta Crystallographica Section C*, 1987, **43**, 456-457.
8. C. Hu, Q. Li and U. Englert, Structural trends in one and two dimensional coordination polymers of cadmium(ii) with halide bridges and pyridine-type ligands, *CrystEngComm*, 2003, **5**, 519-529.
9. K. Biradha and M. Fujita, 2D and 1D Coordination Polymers with the Ability for Inclusion of Guest Molecules: Nitrobenzene, Benzene, Alkoxysilanes, *Journal of inclusion phenomena and macrocyclic chemistry*, 2001, **41**, 201-208.
10. L. Mi, H. Hou, Z. Song, H. Han and Y. Fan, Polymeric Zinc Ferrocenyl Sulfonate as a Molecular Aspirator for the Removal of Toxic Metal Ions, *Chemistry – A European Journal*, 2008, **14**, 1814-1821.
11. S. A. Barnett, A. J. Blake, N. R. Champness and C. Wilson, Structural diversity in two-dimensional coordination polymers constructed from simple building-blocks; a rare example of coordination polymer polymorphs structurally characterised from multiple crystals, *Dalton Transactions*, 2005, 3852-3861.
12. B.-Y. Lou, *Jiegou Huaxue*, 2004, **23**, 747.
13. B. S. J.Pickardt, Z.Naturforsch *B:Chem.Sci* 1996, **51**, 947.
14. M. Lusi, J. L. Atwood, L. R. MacGillivray and L. J. Barbour, Isostructural coordination polymers: epitaxis vs. solid solution, *CrystEngComm*, 2011, **13**, 4311-4313.
15. J. Choi, J. D. Woodward, J. L. Musfeldt, C. P. Landee and M. M. Turnbull, Vibrational Properties of Cu(Pz)2(ClO4)2: Evidence for Enhanced Low-Temperature Hydrogen Bonding in Square S = 1/2 Molecular Antiferromagnets, *Chemistry of Materials*, 2003, **15**, 2797-2802.
16. A. A. Lemus-Santana, J. Rodríguez-Hernández, M. González, S. Demeshko, M. Ávila, M. Knobel and E. Reguera, Synthesis and characterization of T[Ni(CN)4]·2pyz with T=Fe, Ni; pyz=pyrazine: Formation of T–pyz–Ni bridges, *Journal of Solid State Chemistry*, 2011, **184**, 2124-2130.
17. J. Rodríguez-Hernández, A. A. Lemus-Santana, J. Ortiz-López, S. Jiménez-Sandoval and E. Reguera, Low temperature structural transformation in T[Ni(CN)4]·xpyz with x=1,2; T=Mn,Co,Ni,Zn,Cd; pyz=pyrazine, *Journal of Solid State Chemistry*, 2010, **183**, 105-113.
18. W. Clegg, I. R. Little and B. P. Straughan, Monoclinic anhydrous zinc(II) acetate, *Acta Crystallographica Section C*, 1986, **42**, 1701-1703.
19. H. He, A new monoclinic polymorph of anhydrous zinc acetate, *Acta Crystallographica Section E*, 2006, **62**, m3291-m3292.

20. N. Clavier, N. Hingant, M. Rivenet, S. Obbade, N. Dacheux, N. Barré and F. Abraham, X-Ray Diffraction and μ-Raman Investigation of the Monoclinic-Orthorhombic Phase Transition in Th1−xUx(C2O4)2·2H2O Solid Solutions, *Inorganic Chemistry*, 2010, **49**, 1921-1931.
21. X.-y. X. Jian Wang, Wei-xing Ma, Hong Zhao, Lu-de Lu, Xu-jie Yang, Xin Wang *Rengong Jingti Xuebao* 2008, **37**, 1199.
22. S. Shen and L. Zhao, M0.5C11H12N2O2 (M = Zn, Co, Ni): A Two-dimensional Layered Hybrid Compound Derived from L-Tryptophan Containing Helical Chains, *Zeitschrift für anorganische und allgemeine Chemie*, 2011, **637**, 2099-2102.
23. H. Xu, X. Zeng, W. Pan, J. Zhang, Y. Cao, H. Guo and J. Xie, Structural diversity of metal–organic frameworks based on a chalcone dicarboxylic acid ligand, *Dalton Transactions*, 2020, **49**, 5783-5786.
24. P. Jensen, S. R. Batten, G. D. Fallon, B. Moubaraki, K. S. Murray and D. J. Price, Structural isomers of M(dca)2 molecule-based magnets. Crystal structure of tetrahedrally coordinated sheet-like β-Zn(dca)2 and β-Co/Zn(dca)2, and the octahedrally coordinated rutile-like α-Co(dca)2, where dca– = dicyanamide, N(CN)2–, and magnetism of β-Co(dca)2, *Chemical Communications*, 1999, 177-178.
25. R. Marsh, The structure of [CdNi(CN)4(C6H8N2)(NH3)]: Corrigendum, *Acta Crystallographica Section C*, 1989, **45**, 694-695.
26. T. Hokelek and D. Ulku, Structure of catena-poly[tri-[mu]-cyano-(ammine)(2-amino-3-methylpyridine)cadmium-[mu]-cyano-nickel], *Acta Crystallographica Section C*, 1988, **44**, 832-834.
27. Y. Gong, J. Liu, C. Hu and W. Gao, Solvent-induced supramolecular isomers: Two dimensional coordination polymers constructed by Cu(II) and fluconazole, *Inorganic Chemistry Communications*, 2007, **10**, 575-579.
28. S.-S. B. Bei Liu, Min Ren, Zhong-Sheng Cai, Jing-Cui Liu, Li-Min Zheng *Wuji Huaxue Xuebao* 2020, **36**, 1185.
29. Y.Iimura, *Sci.Pap.Inst.P.C.R.(Jpn.)* 1973, **67**, 43.
30. D. W. Tomlin, T. M. Cooper, D. E. Zelmon, Z. Gebeyehu and J. M. Hughes, Cadmium isopropylxanthate, *Acta Crystallographica Section C*, 1999, **55**, 717-719.
31. P. Samarasekere, X. Wang, A. J. Jacobson, J. Tapp and A. Möller, Synthesis, Crystal Structures, Magnetic, and Thermal Properties of Divalent Metal Formate–Formamide Layered Compounds, *Inorganic Chemistry*, 2014, **53**, 244-256.
32. M. L. Hernández, M. G. Barandika, M. K. Urtiaga, R. Cortés, L. Lezama and M. I. Arriortua, Structural analysis and magnetic properties of the 2-D compounds [M(N3)2(bpa)] (M = Mn, Co or Ni; bpa = 1,2-bis(4-pyridyl)ethane)*Journal of the Chemical Society, Dalton Transactions*, 2000, 79-84.
33. C. S. Hong, S.-K. Son, Y. S. Lee, M.-J. Jun and Y. Do, High-Dimensional Manganese(II) Compounds with Noncovalent and/or Covalent Bonds Derived from Flexible Ligands: Self-Assembly and Structural Transformation, *Inorganic Chemistry*, 1999, **38**, 5602-5610.
34. C. Bellitto, F. Federici, M. Colapietro, G. Portalone and D. Caschera, X-ray Single-Crystal Structure and Magnetic Properties of Fe[CH3PO3)]·H2O: A Layered Weak Ferromagnet, *Inorganic Chemistry*, 2002, **41**, 709-714.
35. P. Léone, P. Palvadeau, K. Boubekeur, A. Meerschaut, C. Bellitto, E. M. Bauer, G. Righini and P. Fabritchnyi, Dimorphism in iron(II) methylphosphonate: Low-temperature crystal structure and temperature-dependent Mössbauer studies of a new form of the layered weak ferromagnet Fe[(CH3PO3)(H2O)], *Journal of Solid State Chemistry*, 2005, **178**, 1125-1132.
36. M. R. Montney, R. M. Supkowski, R. J. Staples and R. L. LaDuca, Synthesis, crystal structure, and magnetic properties of two-dimensional divalent metal glutarate/dipyridylamine coordination polymers, with a single crystal-to-single crystal transformation in the copper derivative, *Journal of Solid State Chemistry*, 2009, **182**, 8-17.

37. R. E. Marsh and V. Schomaker, Some incorrect space groups in Inorganic Chemistry, Volume 16, *Inorganic Chemistry*, 1979, **18**, 2331-2336.
38. M. Cannas, G. Carta, A. Cristini and G. Marongiu, Unusual mode of coordination of bis(2-aminoethyl)amine in catena-bis[.mu.-bis(2-aminoethyl)amine]-bis(.mu.-thiocyanato)bis(isothiocyanato)dicadmium(II), *Inorganic Chemistry*, 1977, **16**, 228-230.
39. E. Goldschmied, A. D. Rae and N. C. Stephenson, The crystal structure of ZnII propionate (C6H10O4Zn)n, *Acta Crystallographica Section B*, 1977, **33**, 2117-2120.
40. X.-M. Meng, X.-Y. Zhang, X.-P. Wang, R.-X. Wu, X. Zhang, F. Jin and Y.-H. Fan, Syntheses, crystallization and selective dye adsorption of three Co(II) coordination polymers via one-pot reaction, *Polyhedron*, 2017, **137**, 81-88.
41. V. Martínez, A. B. Gaspar, M. C. Muñoz, G. V. Bukin, G. Levchenko and J. A. Real, Synthesis and Characterisation of a New Series of Bistable Iron(II) Spin-Crossover 2D Metal–Organic Frameworks, *Chemistry – A European Journal*, 2009, **15**, 10960-10971.
42. Y. Xian, S. Y. Niu, J. Jin, L. P. Sun, G. D. Yang and L. Ye, Syntheses, Crystal Structures and Photophysical Properties of a Series of Cadmium(II) Coordination Polymers, *Zeitschrift für anorganische und allgemeine Chemie*, 2007, **633**, 1274-1278.
43. L. P. Nair, B. R. Bijini, R. Divya, P. B. Nair, S. M. Eapen, B. S. Dileep Kumar, S. Nishanth Kumar, C. M. K. Nair, M. Deepa and K. Rajendra Babu, A novel conformation of gel grown biologically active cadmium nicotinate, *Journal of Molecular Structure*, 2017, **1147**, 397-405.
44. V. I. Ovcharenko, G. V. Romanenko, V. N. Ikorskii, R. N. Musin and R. Z. Sagdeev, Polymorphous Modifications of a Ni2+ Complex with Stable Nitroxide Involving Ni2+-O.bul.-N Bonds. Quantum-Chemical Investigation of Exchange Interactions in Heterospin Systems, *Inorganic Chemistry*, 1994, **33**, 3370-3381.
45. J. Navarro-Sánchez, I. Mullor-Ruíz, C. Popescu, D. Santamaría-Pérez, A. Segura, D. Errandonea, J. González-Platas and C. Martí-Gastaldo, Peptide metal–organic frameworks under pressure: flexible linkers for cooperative compression, *Dalton Transactions*, 2018, **47**, 10654-10659.
46. C. Bartual-Murgui, V. Rubio-Giménez, M. Meneses-Sánchez, F. J. Valverde-Muñoz, S. Tatay, C. Martí-Gastaldo, M. C. Muñoz and J. A. Real, Epitaxial Thin-Film vs Single Crystal Growth of 2D Hofmann-Type Iron(II) Materials: A Comparative Assessment of their Bi-Stable Spin Crossover Properties, *ACS Applied Materials & Interfaces*, 2020, **12**, 29461-29472.
47. G. Agustí, M. C. Muñoz, A. B. Gaspar and J. A. Real, Spin-Crossover Behavior in Cyanide-bridged Iron(II)−Gold(I) Bimetallic 2D Hofmann-like Metal−Organic Frameworks, *Inorganic Chemistry*, 2008, **47**, 2552-2561.
48. T. Kosone, C. Kanadani, T. Saito and T. Kitazawa, Synthesis, crystal structures, magnetic properties and fluorescent emissions of two-dimensional bimetallic coordination frameworks FeII(3-fluoropyridine)2[AuI(CN)2]2 and MnII(3-fluoropyridine)2[AuI(CN)2]2, *Polyhedron*, 2009, **28**, 1930-1934.
49. X.-D. Chen, H.-F. Wu, X.-H. Zhao, X.-J. Zhao and M. Du, Metal−Organic Coordination Architectures with Thiazole-Spaced Pyridinecarboxylates: Conformational Polymorphism, Structural Adjustment, and Ligand Flexibility, *Crystal Growth & Design*, 2007, **7**, 124-131.
50. C. Robl, Komplexe mit aromatischen Carbonsäuren. V. Über die Schichtstruktur von Cd[C6H4(COO)2] · H2O, *Zeitschrift für anorganische und allgemeine Chemie*, 1988, **566**, 144-150.
51. J. L. L. Vaz, G. Duc, M. Petit-Ramel, R. Faure and O. Vittori, Cd(II) complexes with phthalic acids: solution study and crystal structure of cadmium(II) phthalate hydrate, *Canadian Journal of Chemistry*, 1996, **74**, 359-364.
52. S. Wang, Y. Hou, E. Wang, Y. Li, L. Xu, J. Peng, S. Liu and C. Hu, A novel organic-inorganic hybrid material with fluorescent emission: [Cd(PT)(H2O)]n (PT = phthalate), *New Journal of Chemistry*, 2003, **27**, 1144-1147.

53. R. Sanii, C. Hua, E. Patyk-Kaźmierczak and M. J. Zaworotko, Solvent-directed control over the topology of entanglement in square lattice (sql) coordination networks, *Chemical Communications*, 2019, **55**, 1454-1457.
54. B. A. Inc, Apex4, 2012.
55. L. Krause, R. Herbst-Irmer, G. M. Sheldrick and D. Stalke, Comparison of silver and molybdenum microfocus X-ray sources for single-crystal structure determination, *Journal of Applied Crystallography*, 2015, **48**, 3-10.
56. G. M. Sheldrick, Crystal structure refinement with<i>SHELXL</i>, *Acta Crystallographica Section C Structural Chemistry*, 2015, **71**, 3-8.
57. A. L. Spek, Single-crystal structure validation with the program<i>PLATON</i>, *Journal of Applied Crystallography*, 2003, **36**, 7-13.
58. T. Degen, M. Sadki, E. Bron, U. König and G. Nénert, The HighScore suite, *Powder Diffraction*, 2014, **29**, S13-S18.
59. S. P. Thompson, J. E. Parker, J. Marchal, J. Potter, A. Birt, F. Yuan, R. D. Fearn, A. R. Lennie, S. R. Street and C. C. Tang, Fast X-ray powder diffraction on I11 at Diamond, *Journal of Synchrotron Radiation*, 2011, **18**, 637-648.
60. A. Altomare, C. Cuocci, C. Giacovazzo, A. Moliterni, R. Rizzi, N. Corriero and A. Falcicchio, EXPO2013: a kit of tools for phasing crystal structures from powder data, *Journal of Applied Crystallography*, 2013, **46**, 1231-1235.
61. B. H. Toby and R. B. Von Dreele, GSAS-II: the genesis of a modern open-source all purpose crystallography software package, *Journal of Applied Crystallography*, 2013, **46**, 544-549.
62. P. E. Blöchl, Projector augmented-wave method, *Physical review B*, 1994, **50**, 17953.
63. G. Kresse and J. Furthmüller, Efficiency of ab-initio total energy calculations for metals and semiconductors using a plane-wave basis set, *Computational materials science*, 1996, **6**, 15-50.
64. G. Kresse and J. Furthmüller, Efficient iterative schemes for ab initio total-energy calculations using a plane-wave basis set, *Physical review B*, 1996, **54**, 11169.
65. J. Wellendorff, K. T. Lundgaard, A. Møgelhøj, V. Petzold, D. D. Landis, J. K. Nørskov, T. Bligaard and K. W. Jacobsen, Density functionals for surface science: Exchange-correlation model development with Bayesian error estimation, *Physical Review B*, 2012, **85**, 235149.
66. H. J. Monkhorst and J. D. Pack, Special points for Brillouin-zone integrations, *Physical review B*, 1976, **13**, 5188.
67. J. D. Pack and H. J. Monkhorst, " Special points for Brillouin-zone integrations"—a reply, *Physical Review B*, 1977, **16**, 1748.
68. D. S. BIOVIA, Material Studio, BIOVIA, Dassault Systèmes, Material Studio.*Journal*, 2022.
69. H. Sun, COMPASS: An ab Initio Force-Field Optimized for Condensed-Phase ApplicationsOverview with Details on Alkane and Benzene Compounds, *The Journal of Physical Chemistry B*, 1998, **102**, 7338-7364.
70. H. Sun, Z. Jin, C. Yang, R. L. C. Akkermans, S. H. Robertson, N. A. Spenley, S. Miller and S. M. Todd, COMPASS II: extended coverage for polymer and drug-like molecule databases, *Journal of Molecular Modeling*, 2016, **22**, 47.
